# Upconversion nanoplatform unlocks the theranostic potential of Lu-177 radiotherapy via c-MET axis targeting in head and neck squamous cell carcinoma

**DOI:** 10.1016/j.mtbio.2026.103585

**Published:** 2026-08-25

**Authors:** Kai-Hung Lin, Chun-Yi Wu, Yu-Chan Chang, Ming-Hsien Chan

**Affiliations:** Department of Biomedical Imaging and Radiological Sciences, National Yang Ming Chiao Tung University, Taipei, 112, Taiwan

**Keywords:** Upconversion nanoparticles, Lu-177 therapy, c-Met pathway, Radiological therapy, SPECT imaging

## Abstract

Upconversion nanoparticles (UCNPs) convert tissue-penetrating near-infrared photons into sharp visible emissions, offering background-free optical readouts for deep-tissue imaging. Here we report multilayer NaLuF_4_:Yb,Tm@NaLuF_4_ UCNPs that simultaneously act as fluorescent probes and radioisotope precursors. Under 980 nm excitation, the particles generate bright blue and red upconversion signals for real-time tracking. At the same time, their lutetium-rich core can be neutron-activated in a single step to yield β^−^- and γ-emitting ^177^Lu. The β-particles confine cytotoxic dose within a 2 mm range, whereas the co-emitted γ photons enable quantitative single-photon emission computed tomography (SPECT). To enhance the tumor selectivity of the nanoplatform, we grafted a c-Met DNA aptamer onto the PEGylated shell, guiding the nanoconstructs to target head and neck squamous cell carcinoma (HNSCC) with amplified c-Met receptor levels. The aptamer markedly enhances cellular uptake, improves therapeutic indices, and limits off-target irradiation. Moreover, the optical modality remains non-radioactive, allowing pre-treatment imaging without patient exposure, and supplements the relatively low (∼10%) γ yield of ^177^Lu for accurate intra-procedural localization. Collectively, this single nanosystem unites targeted radiotherapy, radionuclide therapy, SPECT, and NIR-mediated optical imaging, delivering a coherent “see-and-treat” strategy for HNSCC. The modular design also provides a versatile platform that can be transferred to other solid tumors bearing actionable biomarkers across diverse clinical oncology imaging and therapy settings.

## Highlights

• A dual-function UCNP platform integrates optical imaging and radioisotope generation.
• Single-step neutron activation enables *in situ* production of ^177^Lu for SPECT imaging and radiotherapy.
• Aptamer-guided tumor selectivity that enhances uptake and therapeutic efficacy while minimizing collateral dose.
• A “see-and-treat” nanoplatform integrates tumor visualization with targeted radionuclide therapy.

## 1 Introduction

**Scheme 1:**
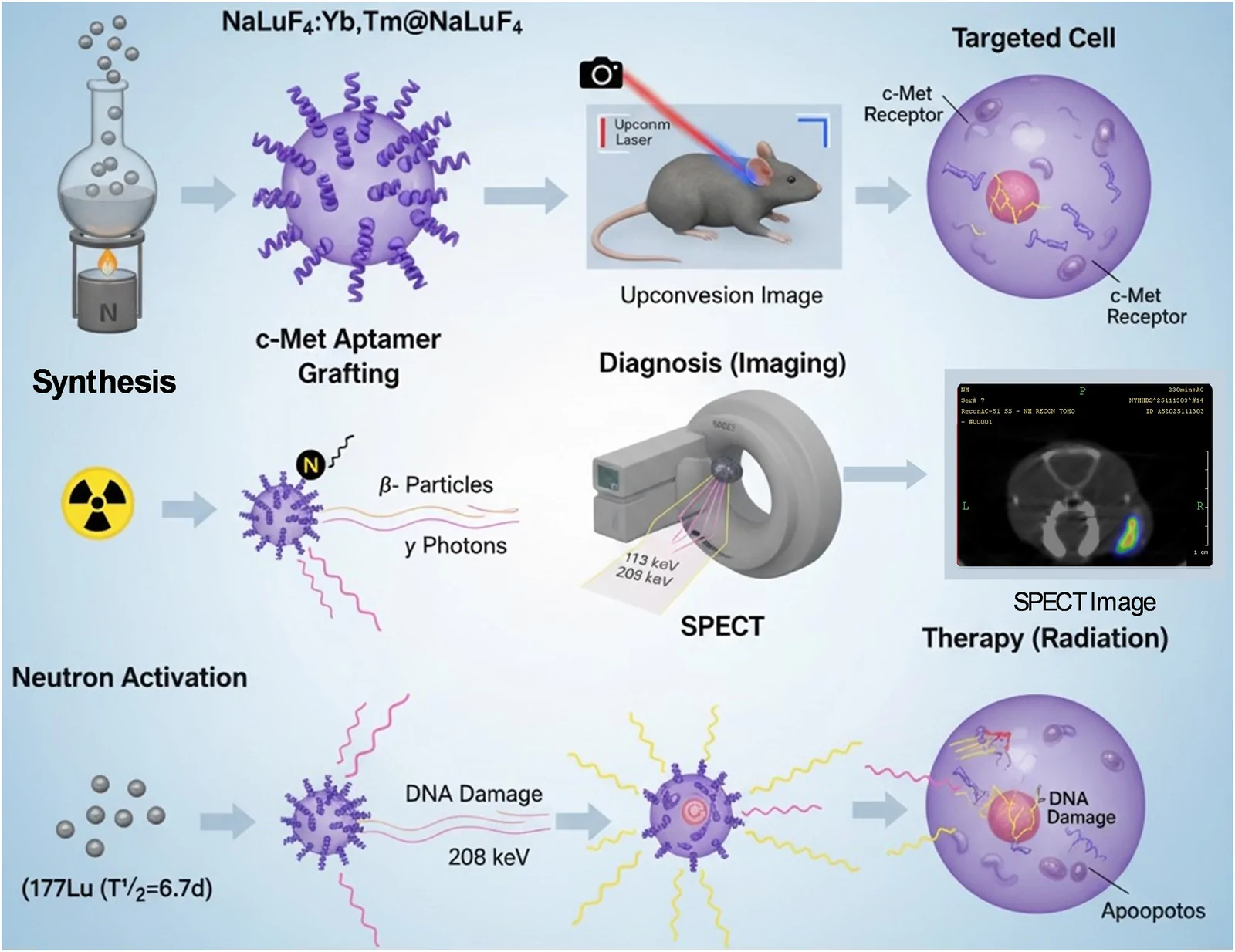
Schematic diagram of the experimental preparation of a dual-modal system of radioactive Lutetium-based upconversion nanoparticles for application in cancer diagnosis and treatment.

Upconversion nanoparticles (UCNPs) are a class of lanthanide-doped nanomaterials that convert low-energy near-infrared (NIR) light into higher-energy visible or ultraviolet light through upconversion luminescence (UCL) [1]. This unique anti-Stokes emission mechanism relies on the sequential absorption of multiple photons, primarily mediated by sensitizer ions such as Yb^3+^ and by activator ions such as Tm^3+^, Er^3+^, or Ho^3+^ [2]. Among the many host matrices developed, NaLuF_4_-based UCNPs are recognized for their efficient luminescence, chemical stability, and biocompatibility [3]. When excited by a 980 nm NIR laser, these nanoparticles emit sharp, intense visible signals with minimal background autofluorescence and deeper tissue penetration than traditional fluorophores, making them ideal for biological imaging, drug delivery, and light-triggered therapies [4]. In recent years, UCNPs have been functionally extended to include radiological properties, resulting in a new subclass: radioluminescent upconversion nanoparticles [5]. This approach involves integrating radionuclide-generating elements, such as lutetium (Lu), into the nanoparticle matrix. Upon neutron activation (e.g., via Lu-176 + n → Lu-177 + γ rays), the inert nanoparticle is converted into a radiotherapeutic agent [6]. Lutetium-177 (Lu-177) is a clinically approved β^−^ emitting isotope with a half-life of 6.7 days [[7], [8], [9]]. It emits both therapeutic β-particles (∼500 keV, range < 2 mm) and γ photons (113 keV and 208 keV), the latter suitable for single-photon emission computed tomography (SPECT) imaging [10]. This dual capability enables radiological UCNPs to serve as a theranostic platform, combining therapeutic irradiation and diagnostic imaging in a single nanosystem [11] (see Scheme 1)

The integration of optical and radiation imaging functions with site-specific tumor targeting is particularly relevant in the context of head and neck squamous cell carcinoma (HNSCC), a malignancy with high incidence and mortality in South and Southeast Asia [12,13]. Despite being largely preventable, HNSCC continues to pose significant clinical challenges due to delayed diagnosis, aggressive tumor behavior, and limited treatment options. Conventional therapies, including surgery, radiation, and chemotherapy, are often associated with high toxicity and limited specificity [14]. Therefore, there is a compelling need for advanced strategies that enable precise tumor detection and effective therapeutic intervention with minimal systemic side effects [15]. Driver gene activation mutations are relatively rare in HNSCC, but the abnormal activation of the oncogenes epidermal growth factor receptor (EGFR) and mesenchymal-to-epithelial transition factor (c-Met) is almost universal in HNSCC [16]. Activation of the c-Met pathway by its ligand, hepatocyte growth factor (HGF), promotes cancer cell proliferation, motility, invasion, and resistance to apoptosis. Moreover, emerging evidence suggests that c-Met overexpression correlates with poor prognosis and therapeutic resistance, including resistance to epidermal growth factor receptor (EGFR) inhibitors [17,18]. Although cetuximab, an anti-EGFR monoclonal antibody, is available, antibody-drug conjugates (ADCs) for c-Met are still under development and have not yet demonstrated clear efficacy [19].

To address these challenges, the present study proposes a novel UCNP-based radiotheranostic nanoplatform designed for targeted imaging and treatment of HNSCC [20]. Specifically, we developed a core–shell structured UCNP, NaLuF_4_:Yb,Tm@NaLuF_4_, with a lutetium-doped core enabling post-synthesis neutron activation to generate Lu-177, unlike conventional chelator-based radiolabeling strategies, Lu was intrinsically embedded within the nanoparticle lattice, allowing direct neutron activation without additional radiochemical conjugation [21]. The core–shell design enhances upconversion luminescence by minimizing surface quenching and provides a robust structure for stable radiolabeling. The outer shell is modified with polyethylene glycol (PEG) to improve colloidal stability and biocompatibility, while the surface is further conjugated with a c-Met-specific DNA aptamer [22]. Aptamers are short, single-stranded oligonucleotides selected for their high binding affinity and specificity toward target molecules [23]. They offer several advantages over traditional antibodies, including lower immunogenicity, easier synthesis, and improved tumor penetration [24]. Upon neutron activation, the UCNPs emit β^−^ radiation from Lu-177, effectively inducing DNA damage and apoptosis in cancer cells within the targeted tumor site. Simultaneously, the γ emissions enable real-time SPECT imaging, providing precise information about nanoparticle biodistribution [25]. In addition, under 980 nm NIR excitation, the UCNPs emit visible fluorescence, enabling optical tracking of the nanomaterials [26]. This dual-modal imaging, combining optical and nuclear modalities, provides complementary anatomical and functional insights, facilitating image-guided therapy [27]. Using c-Met aptamer functionalization further enhances tumor selectivity by ensuring that the UCNPs preferentially accumulate in c-Met-overexpressing cancer cells. This targeted delivery approach improves the therapeutic index and minimizes off-target effects on healthy tissues [28]. Notably, the non-radioactive version of these nanoparticles exhibits minimal cytotoxicity, supporting their safety profile for *in vivo* applications [29].

In summary, this study presents an innovative strategy that integrates radiotherapy, targeted molecular therapy, and multimodal imaging into a single UCNP-based system for the diagnosis and treatment of HNSCC [30]. By harnessing the unique optical properties of UCNPs, the therapeutic potential of Lu-177, and the targeting capability of c-Met aptamers, we provide a novel platform with high translational potential for personalized oncology. To better clarify the distinction from previous studies, a comparison summarizing previously reported Lu-based UCNP theranostic systems and the present work is provided in Table 1. This theranostic approach holds promise for improving the precision and efficacy of HNSCC treatment and broader applications in other solid tumors with aberrant c-Met signaling.

**Table 1:** Comparison table summarizing previously reported Lu-based UCNP theranostic systems and the present work.

Nanocomposites	Particle size	Diagnostic imaging	Radiotherapy	Active Targeting	Ref.
NaGdF_4_/Ho-Yb@m-SiO_2_	200−300 nm	Single-photon emission computed tomography and upconversion imaging	^177^Lu-labeled nanoparticles	n/a	[31]
NaGdF_4_:Yb@NaErF_4_:Tm@NaYF_4_:Yb/^177^Lu@NaYF_4_@DSPE-PEG-HER2	≈ 90 nm	Up- and down- conversion imaging	^177^Lu-labeled nanoparticles	EGFR-targeting	[32]
^177^Lu-YNP@FA	79 nm	Single-photon emission computed tomography and upconversion imaging	^177^Lu-labeled nanoparticles	Folic acid	[33]
UCNP@mSiO_2_-PFC/Ce6@RAW-Man/PTX	54.1 ± 1.3 nm	Photoacoustic images and upconversion imaging	n/a	Raw 264.7 membrane	[34]
UCNP/Ce6-loaded Hb μGels	∼35 nm	Computerized tomography and upconversion imaging	n/a	n/a	[35]
NaLuF_4_:Yb,Tm@NaLuF_4_-cMet (This work)	∼200 nm	Single-photon emission computed tomography and upconversion imaging	^177^Lu-nanoparticles (No additional radio-labeling required)	c-Met-targeting	-

## 2 Experimental section

### 2.1 Materials and chemicals

All the reagents and chemicals used in the experiment were of analytical grade and were not further purified. Lutetium(III) acetate hydrate (CH_3_CO_2_)_3_Lu · xH_2_O, Ytterbium(III) acetate tetrahydrate Yb(C_2_H_3_O_2_)_3_·4H_2_O (99.9%), Thulium(III) acetate hydrate Tm(CH_3_CO_2_)_3_ · xH_2_O (99.9%), Ammonium fluoride (NH_4_F), Sodium hydroxide (NaOH), Oleic acid (90%), 1-Octadecene 90%, N-hydroxysuccinimide (NHS), α,ω-Bis{2-[(3-carboxy-1-oxopropyl)amino]ethyl}polyethylene glycol, M_n_ ~ 3000), Dicyclohexylcarbodiimide (DCC), Dopamine hydrochloride, Chloroform (CHCl_3_), N,N Dimethylformamide (DMF), Sodium carbonate (Anhydrous Na_2_CO_3_), Hexane, Ethanol, PBS, N-(3-dimethylaminopropyl)-N-ethylcarbodiimide hydrochloride (EDC), the chemicals listed above were all purchased from Sigma–Aldrich. c-Met Aptamer (TGG ATG GTA GCT CGG TCG GGG TGG GTG GGT TGG CAA GTC T) was purchased from Genomics. Deionized water was used wherever water was needed, and methanol was used throughout the experiments.

### 2.2 Synthesis of core-shell upconversion nanoparticles

Upconversion nanoparticles (UCNPs) with a core/shell structure were synthesized using the thermal decomposition method [36]. First, the UCNP core NaLuF_4_:Yb,Tm was synthesized by adding 3.16 mL of Lu(CH_3_CO_2_)_3_·xH_2_O (0.2 M/40 mL), 0.8 mL of Yb(CH_3_CO_2_)_3_·xH_2_O (0.2 M/40 mL), and 0.4 mL of Tm(CH_3_CO_2_)_3_·xH_2_O (0.02 M/40 mL) into a two-neck round-bottom flask. The mixture was then heated to 100°C to evaporate the water for crystallization. Subsequently, 6 mL of OA and 14 mL of ODE were added, followed by a temperature increase to 120°C. The mixture was stirred for 15 min, then the temperature was increased to 170°C and maintained for 1 h before cooling to 60°C.​ In a centrifuge tube, 2 mL of NaOH/MeOH (1 M/40 mL) and 7.9 mL of NH_4_F/MeOH (0.4 M/40 mL) were uniformly mixed and then added to a two-neck round-bottom flask. The flask was then heated to 110°C for 20 min. Vacuum was applied, and nitrogen was introduced to conduct the reaction under a nitrogen atmosphere. The mixture was heated to 300°C for 2.5 h, and then cooled down to room temperature at 45°C. The solution was then retrieved and treated with hexane and ethanol, followed by centrifugation at 8000 RPM for 8 min. This washing process was repeated twice. Finally, the sample was redissolved in hexane and stored. The next step is to synthesize the shell. 2.72 mL of Lu(CH_3_CO_2_)_3_·xH_2_O (0.2 M/40 mL) is added into the two-neck round-bottom flask and heated to 100°C to evaporate the water for crystallization. 6 mL of OA and 14 mL of ODE are added, then heated to 120°C, and the mixture is stirred for 15 min. The temperature was then increased to 170°C and maintained for 1 h before cooling to 60°C. In a centrifuge tube, 2 mL of NaOH/MeOH (1 M/40 mL) and 7.9 mL of NH_4_F/MeOH (0.4 M/40 mL) are mixed uniformly and added to the two-neck round-bottom flask along with the previously synthesized NaLuF_4_:Yb, Tm. The temperature is raised to 110°C and heated for 20 min. Then, a vacuum is applied, and nitrogen is added to maintain the reaction under a nitrogen atmosphere. It is then heated to 300°C for 2.5 h before cooling to 60°C at room temperature. The solution is recovered, treated with hexane and ethanol, and centrifuged at 8000 RPM for 8 min. This washing process is repeated twice. Finally, the sample is redissolved in hexane and stored. The final product obtained is core-shell structured NaLuF_4_:Yb,Tm@NaLuF_4_.

### 2.3 Synthesis of PEG-modified upconversion nanoparticles

Polyethylene glycol (PEG) diacid 3000 (200 mg), NHS (20 mg), Dicyclohexylcarbodiimide (DCC) (30 mg), and dopamine hydrochloride (10.27 mg) were dissolved in a mixed solvent containing CHCl_3_ (20 mL), N,N-Dimethylformamide (DMF) (10 mL), and anhydrous Na_2_CO_3_ (100 mg), and stirred for 2 h. Subsequently, the pre-prepared UCNP (50 mg) was added, and the system was stirred overnight under a nitrogen atmosphere. The modified UCNP was precipitated by adding hexane, and the particles were dispersed in PBS. Excess surfactants and other salts were removed by dialysis using a dialysis bag (molecular weight cut off MWCO = 3000) for 24 h in deionized water. After dialysis, the solution was centrifuged at 8000 rpm for 8 min to remove excess solvent. The PEG-modified UCNP was then redissolved in PBS and stored, named as PEG-UCNP.

### 2.4 Synthesis of aptamer-modified upconversion nanoparticles

Add EDC (8 mg) and NHS (12 mg) to PEG-UCNP (10 mL PBS, 20 mg) solution, and gently shake for 10 min. Then, add 400 nM of c-Met aptamer (TGG ATG GTA GCT CGG TCG GGG TGG GTG GGT TGG CAA GTC T) to 100 mL nuclease-free water and stir at 50 rpm for 4 h. Remove unreacted aptamer by centrifugation at 12000 rpm for 10 min to obtain aptamer-conjugated UCNP. The aptamer-conjugated UCNP was then redissolved in PBS and stored, named cMApt-UCNP.

### 2.5 Using neutrons to stimulate upconversion nanoparticles to become radioactive upconversion nanoparticles

The modification of UCNPs (PEG-UCNP and cMApt-UCNP) was subjected to neutron activation at the Tsing Hua Open Pool Reactor (THOR). The neutron irradiation was performed using a flux of 10^11^−10^12^ neutrons/cm^2^·s for 60–120 min to induce the conversion of Lu-176 into the radioactive isotope Lu-177 [33]. The resulting radioactive UCNPs emitted β-particles for therapeutic applications and γ-rays for SPECT imaging.

### 2.6 Gamma ray spectrometer

The sodium iodide scintillation spectrometer (NaI(Tl)) consists of a thallium-doped sodium iodide crystal, a photomultiplier tube (PMT), and a multi-channel analyzer (MCA). When a γ photon interacts with the NaI(Tl) crystal, its energy is converted into visible scintillation light, which is detected and amplified by the PMT. The MCA then classifies the resulting pulses into energy channels based on pulse height, generating a γ-ray spectrum for both qualitative and quantitative analysis. In this study, a NaI(Tl)-PMT-MCA system was employed to measure the γ-ray energy spectrum of the material, using a dose of 50 μCi and an energy range of 0–500 keV. Spectra were acquired until sufficient counts were accumulated for reliable peak identification and energy calibration.

### 2.7 Half-life examination

An ionization chamber was used to measure and calibrate the radiation dose rate accurately. The chamber, filled with high-purity argon gas and equipped with an anode–cathode electrode pair under a constant electric field, collects ionization currents generated by incident γ-rays, which are amplified by a high-sensitivity electrometer and converted into dose-rate readings. The activity was monitored once daily over 13 days, and the recorded decay data were used to plot the activity–time curve, from which the effective half-life of the radioisotope was calculated.

### 2.8 Gel electrophoresis

Agarose gel electrophoresis was performed to verify the conjugation of the c-Met aptamer to the surface of UCNPs. The nanoparticles were prepared at a final concentration of 250 μg/mL in deionized water and mixed with 6× DNA loading dye at a ratio of 84 μL nanoparticle suspension to 16 μL loading dye. Free c-Met aptamer, AS1411 aptamer (negative control), and cMApt-UCNPs were then loaded onto a 2% agarose gel and separated in 1× TAE buffer at 100 V for 30–40 min. DNA bands were visualized using a UV gel documentation system. Free aptamers migrated to the expected low-molecular-weight region, whereas cMApt-UCNPs remained near the loading wells because of their large particle size, confirming successful conjugation of the c-Met aptamer onto the nanoparticle surface.

### 2.9 Cell lines, culture, and subculturing

OMF, CA922, OC-3, and UM-SCC-1 cells were cultured in Dulbecco's Modified Eagle Medium (DMEM); FaDu, HSC-3, and FaDu-GL in Modified Eagle Medium (MEM); and OECM-1 in RPMI-1640 medium. All media were supplemented with 10% fetal bovine serum (FBS) and 1% penicillin–streptomycin (PS) and maintained in a 37°C incubator with 5% CO_2_. Cells were subcultured at ∼80% confluence. After removing the culture medium, cells were washed 1–2 times with PBS, treated with Trypsin-EDTA for 5–10 min, and neutralized with complete medium. Suspended cells were centrifuged at 1200 rpm for 5 min, resuspended in fresh medium, and seeded into new culture dishes at the desired density for subsequent experiments.

### 2.10 Western blot

Protein expression was analyzed by Western blot. Briefly, cells were scraped from 10-cm dishes, washed with PBS, and lysed in RIPA buffer containing protease inhibitors. Lysates were centrifuged at 1200 rpm, and supernatants were collected and stored at −20°C. Protein concentrations were determined using a BSA standard curve with absorbance measured at 560 nm. Equal amounts of protein (15–30 μg) were mixed with loading dye, denatured at 100°C for 10 min, and separated by 6–10% SDS-PAGE. Proteins were transferred to PVDF membranes and blocked with 4% non-fat milk or 5% BSA in TBST for 1 h at room temperature. Membranes were incubated overnight at 4°C with primary antibodies (1:1000 in 5% BSA), washed, then probed with secondary antibodies (1:10,000 in 4% milk) for 1 h. Signals were detected using chemiluminescent HRP substrate and imaged with the SYNGENE G:Box; band intensities were quantified using ImageJ. For c-Met expression profiling, OMF, CA922, FaDu, OC-3, OECM-1, UM-SCC-1, and HSC-3 cells (1 × 10^6^ cells/dish) were cultured for 48–72 h before protein collection. Primary antibodies used were c-Met (Cell Signaling, D1C2) and β-actin (Genentech, GTX100313). To assess aptamer functionality, FaDu cells were seeded (1 × 10^6^ cells/well, 6-well plates) and treated with PEG-UCNPs or cMApt-UCNPs (500 μg/mL) for 24, 48, or 72 h before protein extraction and analysis with the same primary antibodies. For apoptosis analysis, FaDu cells (1 × 10^6^ cells/well) were treated with radioactive PEG-UCNP + NR or cMApt-UCNP + NR (10^11^–10^12^ neutrons/cm^2^·s for 120 min; ∼500 μg/mL, total 1 mg, ∼100 μCi) and harvested at 6 and 24 h. Blots were probed with caspase-3 (Proteintech, 19677-1-AP), cleaved caspase-3 (Cell Signaling, 5A1E), and GAPDH (Abcam, ab181602) antibodies.

### 2.11 Confocal microscopy

Confocal fluorescence microscopy was used to visualize cellular uptake of nanoparticles. FaDu cells (1 × 10^6^ cells/well) were seeded onto coverslips in 12-well plates and cultured for 24 h. Cells were incubated with PEG-UCNP or cMApt-UCNP (100 μg/mL) for 12 h. To evaluate receptor-mediated uptake, a competitive binding assay was performed by pre-incubating cells with free c-Met aptamer (100 or 300 nM) for 4 h, followed by incubation with cMApt-UCNP (150 μg/mL) for an additional 12 h. After treatment, the cells were washed twice with PBS and fixed with 4% paraformaldehyde (37°C, 10 min), followed by cold methanol (−20°C, 5 min). After three PBS washes, nuclei were counterstained with DAPI mounting medium, and the coverslips were mounted onto glass slides. Images were acquired using a confocal fluorescence microscope equipped with a 980 nm laser for UCNP upconversion fluorescence (500–700 nm emission) and a Cy5 channel (Ex/Em: 646/662 nm). Images were analyzed using LAS X software (version 1.4.6_28433).

### 2.12 TUNEL assay with confocal microscopy

FaDu cells (1 × 10^6^ cells/well) were seeded on coverslips in 12-well plates and cultured for 24 h, followed by treatment with PEG-UCNP or cMApt-UCNP (100 μg/mL) for 12 h. Cells were washed with PBS, fixed with 4% paraformaldehyde (37°C, 10 min), permeabilized with cold methanol (−20°C, 5 min) and 0.1% Triton X-100/0.1% sodium citrate (on ice, 5 min), and washed with PBS. TUNEL reagent (100 μL) was added, and cells were incubated at 37°C for 1 h. After PBS washes, cells were blocked with 2% BSA (1 h, room temperature or overnight), counterstained with DAPI, and mounted on slides. TUNEL fluorescence was imaged and analyzed using LAS X software (v1.4.6_28433).

### 2.13 Flow cytometry

Flow cytometry was used to assess the cellular uptake of PEG-UCNPs and Cy5-labeled cMApt-UCNPs. Subcultured FaDu cells (1 × 10^6^ cells/well) were seeded in 6-well plates and cultured for 24 h. For the standard uptake experiment, cells were treated with PEG-UCNPs or Cy5-labeled cMApt-UCNPs (500 μg/mL) for 9 h. For the competitive binding assay, cells were pre-incubated with free c-Met aptamer (100 or 300 nM) for 4 h, followed by incubation with Cy5-labeled cMApt-UCNPs (150 μg/mL) for an additional 9 h. After incubation, cells were washed twice with PBS, harvested, and resuspended in PBS containing 10% FBS. Cy5 fluorescence (Ex/Em: 650/670 nm) was analyzed by flow cytometry to quantify nanoparticle internalization.

### 2.14 Cell colony formation

FaDu cells (1.5 × 10^6^) were seeded in 10 cm dishes and cultured for 24 h, then treated with PEG-UCNP, cMApt-UCNP, PEG-UCNP + NR, or cMApt-UCNP + NR (100 μg/mL; ∼100 μCi) for 12 h, washed with PBS, and cultured for another 12 h. Cells were then reseeded at 10,000 cells/well in 6-well plates and incubated for 4 days. Colonies were fixed with 4% paraformaldehyde or 10% formalin for 15 min, stained with crystal violet (methanol:acetic acid = 1:3 with 0.4 g crystal violet), washed with PBS, and analyzed using ImageJ.

### 2.15 Animal experiment flow chart

All animal procedures were approved by the Institutional Animal Care and Utilization Committee of National Yang Ming Chiao Tung University (IACUC no. 1140102). Nude mice (immunodeficient, lacking mature T cells due to a Foxn1 gene mutation) were purchased from the National Center for Biological Model Research. Tumor models were established by subcutaneously injecting 5 × 10^5^ FaDu-GL cells into the right buccal region. When tumors reached 80–120 mm^3^, *in vivo* fluorescence imaging was performed at 12, 24, and 48 h post-intravenous injection to confirm tumor accumulation via the EPR effect. Neutron-activated nanoparticles (40 mg/kg, ∼100 μCi) were then administered intravenously for therapy. Tumor volume and body weight were monitored every 3 days. At the end of the study, major organs (heart, liver, spleen, lungs, kidneys, and brain) were harvested, fixed in formalin, and processed for histological analysis.

### 2.16 SPECT/CT acquisition

Imaging was performed on a NanoSPECT/CT Mediso small-animal scanner. Mice were anesthetized (e.g., isoflurane) and positioned supine. A low-dose CT scan was acquired for anatomical reference, followed by a SPECT acquisition. Male nude mice with orthotopic buccal tumors were used for *in vivo* imaging studies. Two mice with closely matched body weights (21.1 g and 21.7 g) were selected and randomly assigned to two groups: PEG-UCNPs (non-targeted) and cMApt-UCNPs (targeted). Each mouse was injected with 500 μCi (≈18.5 MBq) of neutron-activated PEG-UCNPs or cMApt-UCNPs in a volume of 200 μL via the tail vein. The total acquisition time per mouse was chosen to optimize signal-to-noise ratio while keeping anesthesia time acceptable. For SPECT settings, a multi-pin-hole collimator, energy windows centered on the ^177^Lu photopeak (∼208 keV) with appropriate scatter windows, an acquisition matrix appropriate for mouse scale, and projection counts selected to ensure adequate counts given the 700 μCi doses.

### 2.17 Histology and immunohistochemistry

FaDu xenograft tumors were excised 24–48 h after the final treatment and immediately fixed in 10% neutral-buffered formalin for 24 h at room temperature, followed by graded ethanol dehydration, xylene clearing, and paraffin embedding. Sections of 4–5 μm thickness were cut and mounted on positively charged slides, which were baked at 60°C for 1 h before staining. For hematoxylin and eosin (H&E) staining, slides were deparaffinized in xylene, rehydrated through a graded series of ethanol to water, and stained with hematoxylin for 3–5 min. They were then rinsed in tap water and blued in 0.1% ammonia water before counterstaining with eosin for 1–2 min. After dehydration in graded ethanol and clearing in xylene, slides were mounted with a resinous medium and imaged under bright-field microscopy. For immunohistochemistry (IHC), sections were deparaffinized, rehydrated, and subjected to antigen retrieval using Tris-EDTA buffer (pH 9.0) for Ki-67 or citrate buffer (pH 6.0) for cleaved caspase-3 at 95–98°C for 15–20 min, followed by cooling to room temperature. Endogenous peroxidase activity was quenched with 3% hydrogen peroxide for 10 min, and nonspecific binding was blocked with 5% normal serum for 30 min. Slides were then incubated overnight at 4°C with primary antibodies against Ki-67 (1:200–1:500) or cleaved caspase-3 (1:200–1:400), followed by incubation with HRP-conjugated polymer secondary antibodies for 30 min at room temperature. Visualization was achieved using the diaminobenzidine (DAB) chromogen, and sections were counterstained with hematoxylin, dehydrated, cleared, and then mounted. Images were acquired under identical exposure settings at both low and high magnifications (inset scale bar: 2 mm; enlarged scale bar: 200 μm). Quantification of Ki-67 was performed by calculating the percentage of Ki-67-positive nuclei relative to total nuclei, whereas cleaved caspase-3 expression was evaluated as the percentage of positive cells or DAB-stained area. At least five randomly selected fields per tumor section were analyzed in a blinded manner using ImageJ with color deconvolution. Data were reported as mean ± SEM from at least three independent tumors per group, and statistical analysis was performed by one-way ANOVA with Tukey's post hoc test, with significance levels indicated as *p < 0.05, **p < 0.01, and ***p < 0.001.

## 3 Results and discussion

### 3.1 Synthesis and characterization of cMApt-UCNPs

**Fig. 1:**
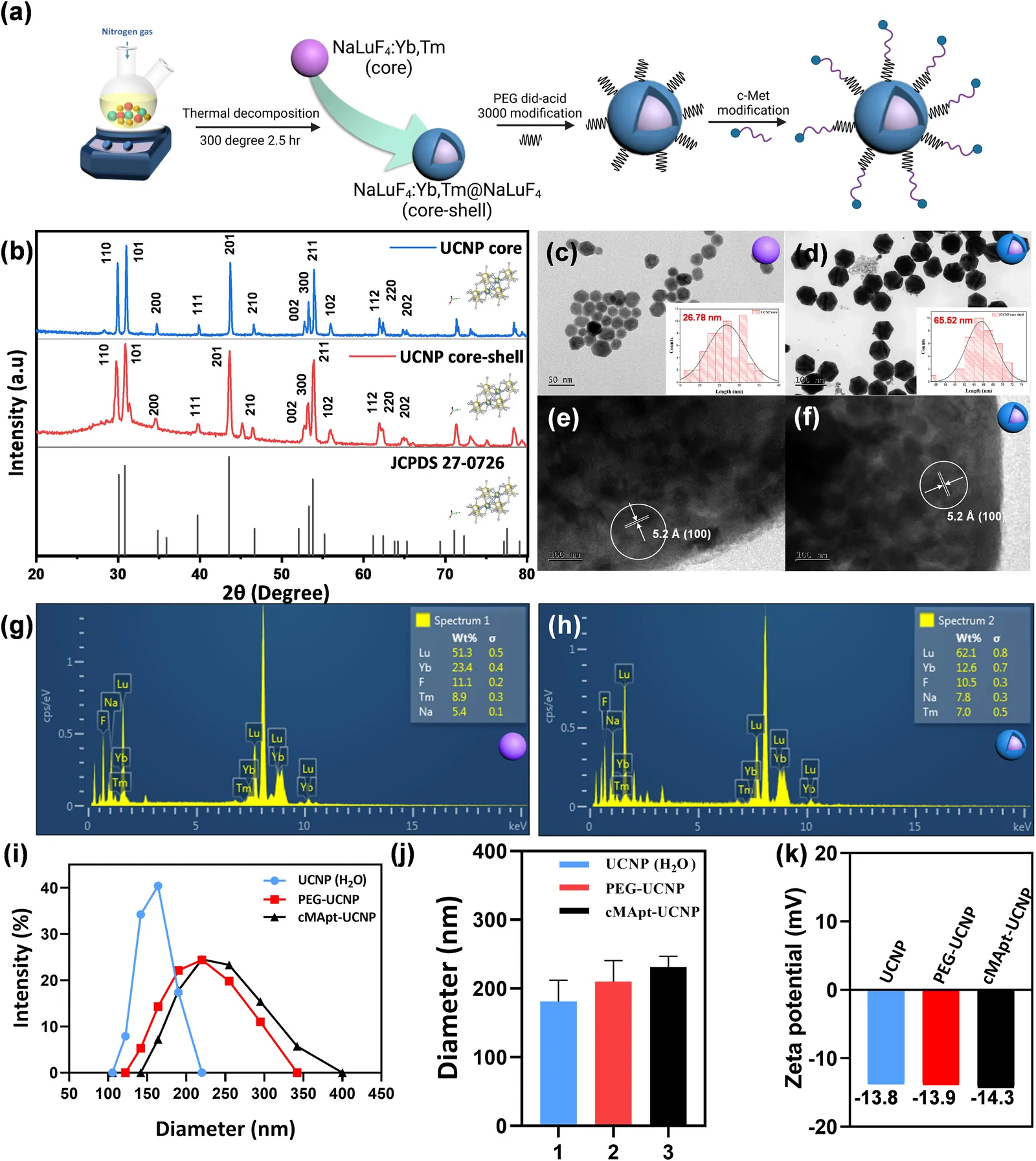
**Basic characterization of Lu-177 UCNP nanoparticles**. (a) Schematic illustration of the synthetic process. (b) XRD pattern of NaLuF_4_:Yb,Tm@NaLuF_4_ compared with the standard reference (JCPDS No. 27-0726), confirming the hexagonal phase. TEM images of nanoparticles at different synthesis stages: (c) Core NaLuF_4_:Yb,Tm (inset: particle size distribution; scale bar: 50 nm) and (d) Core–shell NaLuF_4_:Yb,Tm@NaLuF_4_ (inset: particle size distribution; scale bar: 100 nm). High-resolution TEM images of (e) NaLuF_4_:Yb,Tm and (f) NaLuF_4_:Yb,Tm@NaLuF_4_ showing distinct lattice fringes with an interplanar spacing of 5.2 Å, indexed to the (100) plane of the hexagonal phase, indicating high crystallinity. EDX spectra of (g) NaLuF_4_:Yb,Tm, and (h) NaLuF_4_:Yb,Tm@NaLuF_4_ confirming the elemental composition. (i) DLS measurement showing the size distribution of nanoparticles. (j) Hydrodynamic radius of UCNPs (180.6 ± 18.0 nm), PEG-UCNPs (209.9 ± 17.6 nm), and cMApt-UCNPs (230.8 ± 9.2 nm). (k) Zeta potentials of UCNPs (−13.8 mV), PEG-UCNPs (−13.9 mV), and cMApt-UCNPs (−14.3 mV).

As shown in Fig. 1a, this study used a high-temperature oil-phase pyrolysis method to prepare NaLuF_4_:Yb,Tm@NaLuF_4_ core/shell structure upconversion nanoparticles. Firstly, Lu^3+^, Yb^3+^, and Tm^3+^ were coordinated and pre-crystallized in the organic phase using oleic acid (OA) and 1-Octadecene (ODE) ligand systems, and then a fluorine source (NH_4_F/NaOH) was added, and the crystal nuclei were pyrolyzed at 300°C under nitrogen protection. Finally, core-shell UCNPs with uniform morphology and high luminescence efficiency were obtained via organic-phase precipitation and multiple washings. To achieve the phase transition of UCNPs synthesized in the oil phase to the aqueous phase and improve biocompatibility, a strategy involving the “anchoring” of dopamine to PEG diacid was adopted. The activated PEG-NHS-dopamine was mixed with the UCNPs organic phase suspension, so that the catechol group at the end of dopamine was anchored on the surface of the nanoparticles through coordination. At the same time, the PEG segment provided a hydrophilic barrier. After precipitation, dialysis, and centrifugal purification, PEG-UCNPs were obtained in a stable dispersion in PBS. The retained carboxyl groups on the surface of PEG-UCNPs were chemically activated to amide-activated esters using EDC/NHS, and the resulting esters were then coupled to the 5′-amino-modified c-Met aptamer under mild conditions. After removing the free aptamer by centrifugation, a UCNP-aptamer complex with uniform surface coverage and c-Met receptor targeting was obtained, which can be used for highly selective tumor-targeted imaging and therapy.

In the X-ray diffraction (XRD) analysis, measurements were performed at 40 kV and 25 mA, with a scanning range of 10° to 80°. The shell upconversion nanoparticles (NaLuF_4_:Yb,Tm@NaLuF_4_) synthesized in this study exhibit a hexagonal phase, and their main structure is consistent with the standard spectrum of NaLuF_4_:Yb,Tm in the Joint Committee on Powder Diffraction Standards database (JCPDS 27-0726). As shown in Fig. 1b, the NaLuF_4_:Yb,Tm@NaLuF_4_ synthesized in this study has a hexagonal crystal structure, and its hydrophilicity and target aptamer will be confirmed by subsequent material identification. To achieve uniform hexagonal β-NaLuF_4_:Yb,Tm UCNPs, we systematically explored and optimized various reaction conditions, including precursor ratios, reaction temperature, and growth time, ultimately obtaining well-defined β-phase nanoparticles with high crystallinity (Fig. S1). Transmission electron microscopy (TEM) was used to observe the size and morphology of the nanoparticles. As shown in Fig. 1c and d, both NaLuF_4_:Yb,Tm and NaLuF_4_:Yb,Tm@NaLuF_4_ nanoparticles exhibited good dispersion and uniform hexagonal morphology. Based on the results, the particle size of NaLuF_4_:Yb,Tm was approximately 26.78 nm, while that of NaLuF_4_:Yb,Tm@NaLuF_4_ was approximately 65.52 nm. Particle size analysis was performed using ImageJ software. Moreover, TEM revealed well-defined lattice fringes, with an interplanar spacing (d-spacing) of 5.2 Å, which can be indexed to the (100) plane of hexagonal phase NaLuF_4_, further confirming the high crystallinity of the nanoparticles (Fig. 1e and f). This result is consistent with the XRD analysis, supporting the successful formation of the UCNP hexagonal phase [37].

Energy-dispersive X-ray microanalysis (EDX) is used to analyze the atomic weight ratios of sodium, lutetium, ytterbium, thulium, and fluorine in UCNP nanomaterials; however, this data can only be used for qualitative analysis of the material, not quantitative analysis. As shown in Fig. 1g and h, according to the study, it can be found that the weight percentage of lutetium atoms is 51.3%, the weight percentage of ytterbium atoms is 23.4%, the weight percentage of fluorine atoms is 11.1%, the weight percentage of thulium atoms is 8.9% and the weight percentage of sodium atoms is 5.4%, proving the successful synthesis of NaLuF_4_:Yb,Tm and the weight percentage of lutetium atoms is 62.1%, the weight percentage of ytterbium atoms is 12.6%, the weight percentage of fluorine atoms is 10.5%, the weight percentage of thulium atoms is 7.8% and the weight percentage of sodium atoms is 7.0%, proving the successful synthesis of NaLuF_4_:Yb,Tm@NaLuF_4_. The results show that adding a NaLuF_4_ shell will also increase the total Lu content. Such a core–shell structure not only protects the NaLuF_4_:Yb,Tm core from crystal-lattice damage during subsequent modifications, but also increases the overall Lu content, thereby facilitating its application in radiotherapy.

Dynamic light scattering (DLS) was used to determine the hydrodynamic radius and particle size distribution of nanoparticles at various stages of surface modification. As shown in Fig. 1i, the full distribution curves illustrate the hydrodynamic size distributions of unmodified UCNPs, PEG-UCNPs, and cMApt-UCNPs. The unmodified UCNPs exhibit a size range of approximately 100–220 nm, while PEG-UCNPs display a broader distribution from about 125 to 350 nm. After further conjugation with the c-Met aptamer, cMApt-UCNPs show a size range of approximately 145–400 nm. Notably, the modified samples exhibit tighter, more uniform size distributions, which are attributed to the dialysis purification process. A gradual increase in particle size is observed with each successive surface modification step. Furthermore, the hydrodynamic radius, as shown in Fig. 1j, is approximately 180.6 ± 18.0 nm for the unmodified UCNPs. Following polyethylene glycol modification, the particle size increases to 209.9 ± 17.6 nm, indicating successful PEG coating on the nanoparticle surface. Further conjugation with the c-Met aptamer (cMApt-UCNPs) results in a hydrodynamic radius of 230.8 ± 9.2 nm, representing a modest increase compared to PEG-UCNPs while maintaining good size uniformity (Table S1). Overall, the hydrodynamic radii of all formulations remain below 500 nm in aqueous conditions, indicating that the particle sizes are suitable for *in vivo* mouse experiments.

In this study, the zeta potential was used to analyze the surface potential of the materials. As shown in Fig. 1k, the potentials of UCNP, PEG-UCNP, and cMApt-UCNP are −13.8, −13.9, and −14.3 mV, respectively. The UCNPs are slightly negatively charged after acid washing, which leaves hydroxyl (–OH) groups on their surfaces. For hydrophilic modification, PEG diacid (HOOC–PEG–COOH) was used as the ligand. Only one terminal –COOH group reacts with dopamine to functionalize the UCNPs, while the other free –COOH group remains on the surface. In aqueous solution, this free –COOH can ionize to –COO^-^, contributing to the negative Zeta potential of PEG-UCNPs. Furthermore, the targeting ligand is a small-molecule DNA aptamer. DNA aptamers are inherently negatively charged in neutral environments (such as PBS at pH 7.4) due to the phosphate backbone of each nucleotide (–PO_4_^-^). Therefore, after conjugating the aptamer to PEG-UCNPs, the overall surface charge becomes more strongly negative, resulting in a further decrease in the Zeta potential (i.e., becoming more negatively charged). It can be observed that the materials are slightly negatively charged, which is favorable because they are less likely to aggregate, thereby benefiting subsequent *in vivo* tests.

### 3.2 Optical characterization for precision diagnosis

**Fig. 2:**
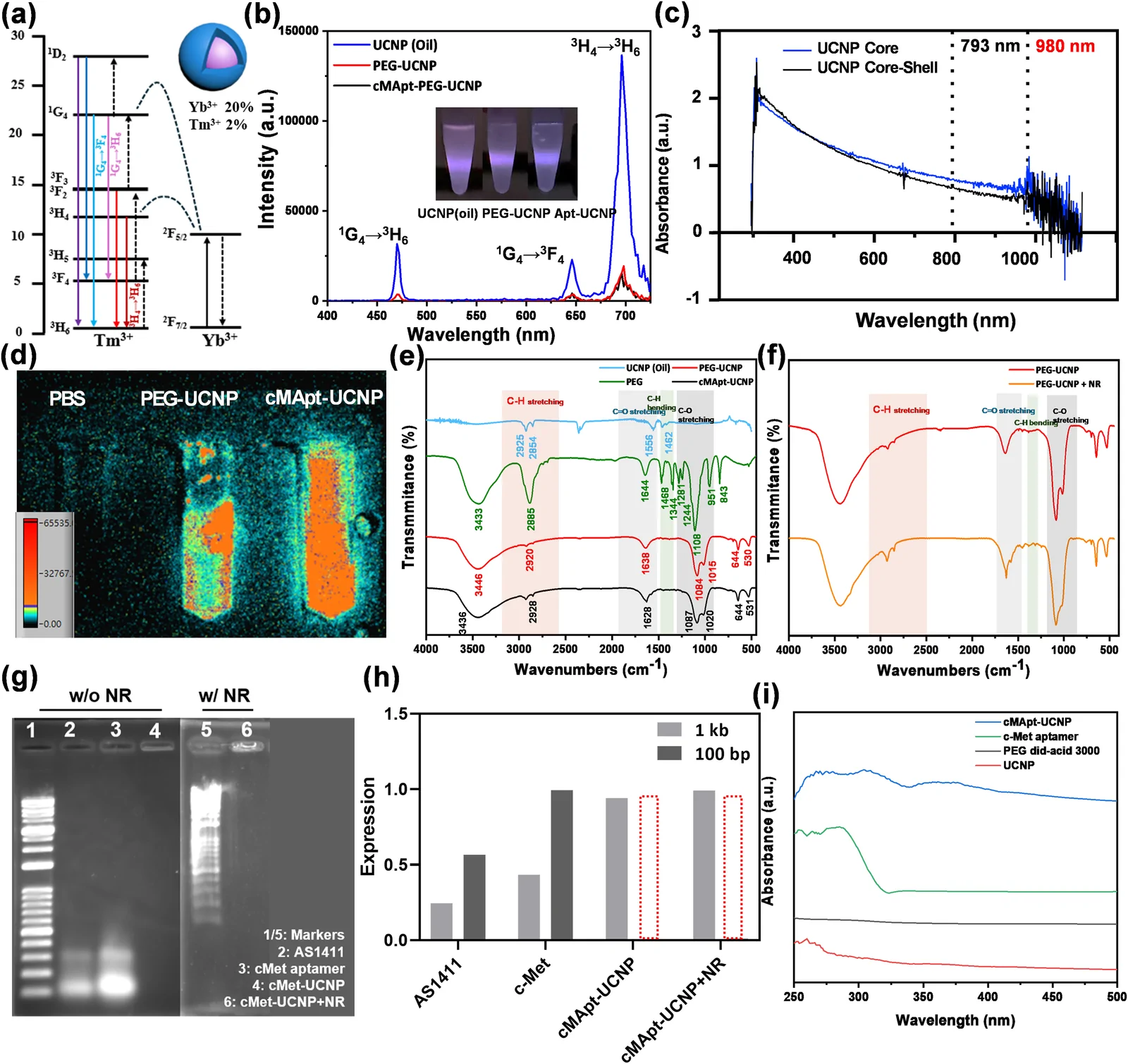
**Optical characterization of UCNPs**. (a) Schematic energy-level diagram of Yb^3+^/Tm^3+^ ions in NaLuF_4_ UCNPs, illustrating the upconversion processes under 980 nm excitation. (b) Upconversion emission spectra of UCNPs, PEG-UCNPs, and cMApt-UCNPs (5 mg/mL) under 980 nm laser excitation, showing characteristic visible emissions at ∼475, 650, and 700 nm. (c) UV–Vis absorption spectrum of UCNPs, displaying a characteristic absorption band corresponding to the Yb^3+^ under 980 nm irradiation. (d) Stability test of UCNP upconversion luminescence under continuous 980 nm laser excitation (20 W, 40%). (e) FTIR spectra of UCNPs, PEG diacid 3000, PEG-UCNPs, and cMApt-UCNPs. (f) FTIR spectra of PEG-UCNPs and PEG-UCNPs loaded with Nile Red (NR). (g) DNA gel electrophoresis results: lane 1 and 5, Markers; lane 2, AS1411 aptamer; lane 3, c-Met aptamer; lane 4, cMApt-UCNPs; lane 6, cMApt-UCNP + NR, (i) UV absorbance spectra of PEG diacid 3000, PEG-UCNPs, and cMApt-UCNPs. (For interpretation of the references to color in this figure legend, the reader is referred to the Web version of this article.)

A fluorescence spectrometer was used to evaluate the upconversion fluorescence of UCNPs under 980 nm laser excitation. UCNP, PEG-UCNP, and cMApt-UCNP were prepared at a concentration of 5 mg/mL, and their emission spectra were recorded, as shown in Fig. 2a and b. The materials exhibited visible-light emissions at approximately 475 nm, 650 nm, and 700 nm. A gradual decline in fluorescence intensity was observed with surface modification. This reduction is primarily attributed to enhanced surface quenching caused by high-energy vibrational groups (–OH, –NH_2_) introduced through PEG and aptamer conjugation, which promote non-radiative relaxation of excited lanthanide ions. Moreover, surface coatings may hinder energy transfer between Yb^3+^ and Tm^3+^, and polymer layers can scatter or absorb excitation and emission light. The transfer from the organic to the aqueous phase during modification also introduces quenching molecules, such as water. Despite reduced luminescence, surface modifications are crucial for enhancing water solubility, biocompatibility, and targeting, which are essential for *in vivo* applications. In addition, UV–Vis absorption spectroscopy confirmed that UCNPs exhibit a characteristic absorption band around 980 nm, corresponding to the ^2^F_7/2_ → ^2^F_5/2_ electronic transition of Yb^3+^ ions, which enables efficient excitation for upconversion luminescence (Fig. 2c). This study used the Vis-NIR real-time imaging detection system of our laboratory to perform upconversion fluorescence imaging. The camera on this machine has a spectral range from visible to near-infrared, and the original excitation light is 980 nm (20W). Therefore, this study used a 980 nm laser to excite the material, enabling the observation of upconversion fluorescence, as shown in Fig. 2d. From left to right, the samples are PBS, PEG-UCNP, and cMApt-UCNP. It was found that PEG-UCNP and cMApt-UCNP can indeed generate upconversion fluorescence with a wavelength of 700 nm under 980 nm laser irradiation.

Additionally, it was found that cMApt-UCNP exhibits the best water dispersibility and is more dispersible than PEG-UCNP in PBS. To further evaluate the fluorescence intensity distribution, quantitative intensity profile analysis was performed (Fig. S2). The results confirmed that cMApt-UCNPs exhibited higher and more uniform fluorescence intensity than PEG-UCNPs under identical excitation conditions. To evaluate the photostability of upconversion nanoparticles (UCNPs), fluorescence imaging was performed with continuous 980 nm laser excitation (20 W, 80%) over 120 min. PEG-UCNP (Fig. S3b) and cMApt-UCNP (Fig. S3a in PBS and Fig. S3b in agarose) exhibited strong and stable upconversion fluorescence at a wavelength of 700 nm at all measurement times (0, 10, 20, 30, 60, and 120 min), with no significant decrease in signal intensity. In contrast, no background signal was observed in the PBS group upon laser excitation, confirming the absence of autofluorescence under the experimental conditions. Furthermore, as shown in Fig. S3c, neutron-activated cMApt-UCNPs (cMApt-UCNP + NR) also maintained stable upconversion luminescence throughout the 120-min continuous laser irradiation period, with no obvious attenuation of fluorescence intensity compared with the non-activated nanoparticles. These results demonstrate that neutron activation did not compromise the photostability of the UCNPs under prolonged 980 nm laser excitation. The consistent fluorescence over time demonstrated that the synthesized UCNPs have excellent photostability and non-photobleaching properties. Unlike traditional organic dyes or quantum dots, which are easily degraded under long-term irradiation, UCNPs maintain their luminescence due to their anti-Stokes excitation mechanism and high chemical stability. This property is particularly advantageous for long-term *in vivo* imaging and real-time monitoring applications.

FTIR spectroscopy was employed to confirm the surface modification of UCNPs at each functionalization step and to evaluate the stability of PEG bonds after neutron irradiation. As shown in Fig. 2e, the spectrum of unmodified UCNPs (OAm-capped) displays characteristic peaks at 2854 cm^−1^ and 2925 cm^−1^, corresponding to the symmetric and asymmetric C–H stretching vibrations from surface-bound oleic acid. After PEG modification, distinct absorption bands appear at 1638 cm^−1^, 1084 cm^−1^, and 1015 cm^−1^, attributed to C=O and C–O stretching vibrations, indicating successful PEGylation of the nanoparticle surface. A broad band around 3446 cm^−1^, associated with O–H stretching, is also present in both PEG and PEG-UCNP spectra, likely due to hydroxyl groups or adsorbed water. Following aptamer conjugation, no new distinct peaks are observed in the FTIR spectrum of cMApt-UCNPs. This absence may result from the low surface density of aptamers or spectral overlap with PEG functional groups. Therefore, while FTIR confirms successful PEG modification, the presence of the aptamer is better verified by complementary techniques. To assess the chemical stability of PEG bonds after neutron activation, FTIR analysis was further conducted on PEG-UCNPs before and after neutron irradiation, as shown in Fig. 2f. The irradiated samples (PEG-UCNP + NR) retained all key functional peaks C–H stretching (∼2885 cm^−1^), C=O stretching (∼1638 cm^−1^), C–O stretching (∼1084 and 1015 cm^−1^), and O–H stretching (∼3446 cm^−1^)—with no noticeable shift or intensity change. These results suggest that the PEG covalent bonds remain intact under the neutron flux conditions used, confirming that the surface PEGylation is chemically stable and not disrupted by neutron exposure. In addition to confirmation by FTIR, UV-Vis was used for observation in this study. First, unmodified UCNP, Aptamer, and cMApt-UCNP were tested respectively. As shown in Fig. 2i, the c-Met aptamer showed strong absorbance below 300 nm. In contrast, PEG has the smallest absorbance in this region. After binding, the cMApt-UCNP spectrum showed absorbance extending below 300 nm, similar to that of the free aptamer, confirming that the aptamer was successfully attached to the nanoparticle surface. This spectroscopic evidence suggests that the aptamer binds effectively to the UCNP surface.

Although FTIR and UV–Vis spectroscopy provided preliminary evidence of surface modification, the characteristic signals corresponding to aptamer conjugation were relatively weak and partially overlapped with PEG-related absorption bands. Therefore, additional NanoDrop was performed to further evaluate aptamer modification on the UCNP surface. As shown in Fig. S4a to S4c, D.D Water and PEG-UCNPs exhibited a relatively low concentration (0.58, 7.1 ng/μL), whereas cMApt-UCNPs showed a markedly increased concentration of 89.89 ng/μL after aptamer conjugation, indicating successful attachment of the c-Met aptamer onto the nanoparticle surface. Furthermore, after neutron irradiation, the cMApt-UCNP + NR group still retained a relatively high concentration (66.44 ng/μL), suggesting that a portion of the surface-conjugated aptamer remained preserved following neutron activation.

Agarose gel electrophoresis analysis verified the binding of the c-Met aptamer to UCNPs, as shown in Fig. 2g. Lane 1 and 5: DNA ladders (markers); Lane 2: AS1411 aptamer (sham control); Lane 3: c-Met aptamer; Lane 4: cMApt-UCNPs; Lane 6: neutron-activated cMApt-UCNPs (cMApt-UCNP + NR). The free aptamers (lanes 2 and 3) exhibited distinct bands in the low-molecular-weight region (∼100 bp). In contrast, no visible DNA band was observed in the corresponding region for either cMApt-UCNPs (lane 4) or cMApt-UCNP + NR (lane 6), indicating that the aptamer was immobilized on the nanoparticle surface and unable to migrate freely through the agarose gel. Notably, the absence of a free aptamer band in lane 6 further demonstrates that neutron activation did not induce detectable aptamer degradation or dissociation from the UCNP surface, confirming that the c-Met aptamer remained stably conjugated after neutron irradiation. To further verify this result, Fig. 2h shows the quantitative analysis of the gel electrophoresis intensity of lanes 2, 3, and 4. The fluorescence intensity in lane 4 was significantly reduced in the low-molecular-weight region (∼100 bp) compared with lanes 2 and 3, supporting the conclusion that the aptamer was bound to the nanoparticle and either retained in the pore or hindered from migrating, thus confirming the successful surface modification.

### 3.3 Radiolabeling and decay analysis of neutron-activated UCNPs

**Fig. 3:**
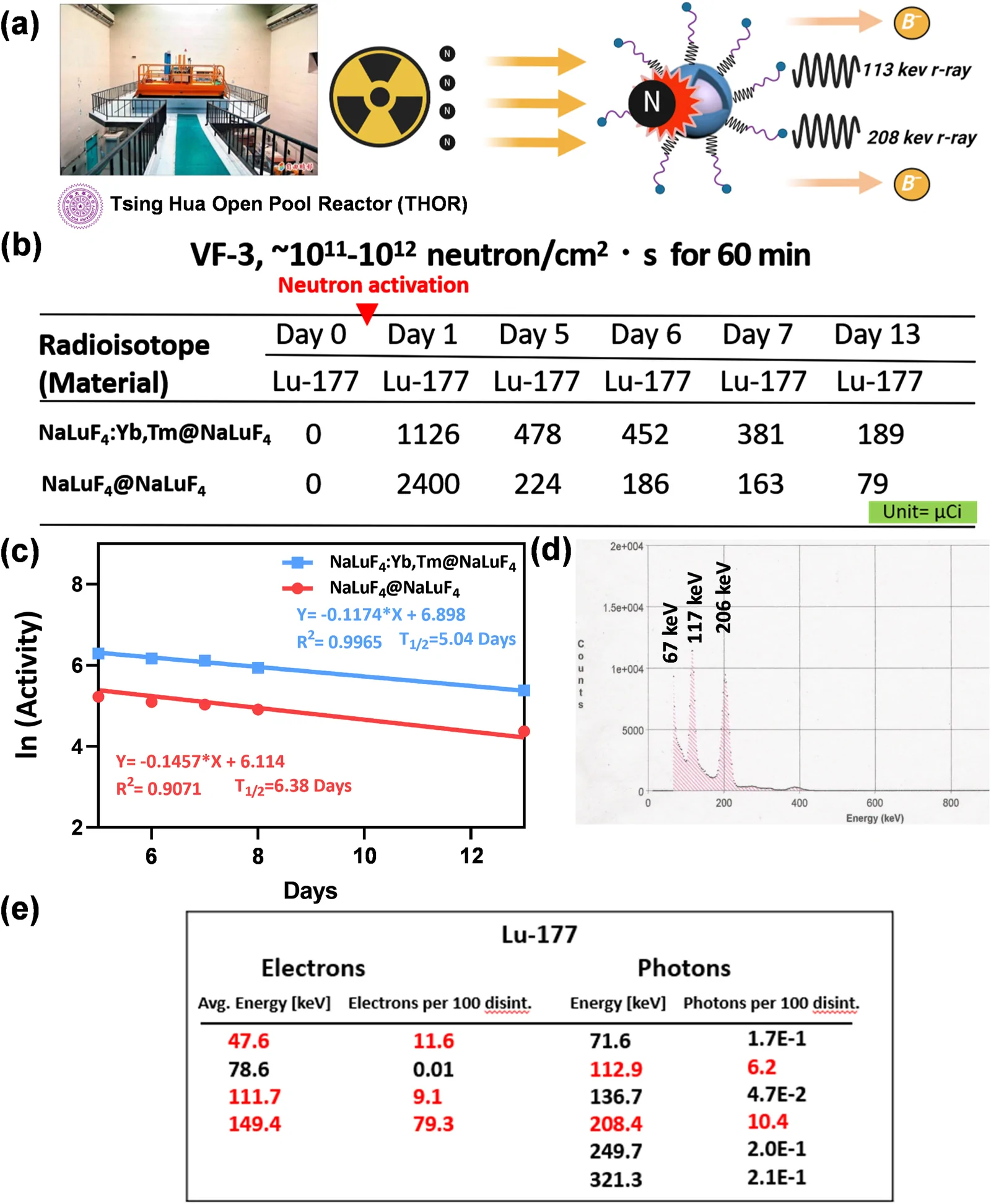
**Characteristics of UCNPs after neutron activation.** (a) Schematic illustration of neutron activation of UCNPs, showing the conversion of stable Lu isotopes to radioactive ^177^Lu. (b) Time-dependent radioactivity measurements and (c) radioactive decay curves of neutron-activated UCNPs. The estimated half-lives of NaLuF_4_:Yb,Tm@NaLuF_4,_ and NaLuF_4_@NaLuF_4_ were 5.04 and 6.38 days, respectively. (d) Gamma-ray spectrum of neutron-activated UCNPs, showing characteristic peaks at 67, 117, and 206 keV. (e) Energy spectrum of the radioactive nuclide lutetium-177 (^177^Lu).

To verify whether the synthesized lutetium-based upconversion nanoparticles (NaLuF_4_:Yb,Tm@NaLuF_4_) successfully produced radioactive ^177^Lu following neutron irradiation, the nanoparticles were irradiated with a flux of approximately 10^11^-10^12^ n/cm^2^·s for 60–120 min to induce the (n,γ) activation (Fig. 3a). All neutron activation procedures were performed at the Tsing Hua Open Pool Reactor (THOR) at National Tsing Hua University, which provides a well-characterized and stable neutron flux suitable for material activation and the generation of radioisotopes. After irradiation, gamma spectroscopy was performed using a multichannel analyzer (MCA) coupled with a scintillator detector. As shown in Fig. 3d, distinct gamma peaks were observed at 67, 117, and 208 keV, with the 113 keV and 208 keV peaks closely matching the known emission energies of ^177^Lu. These results confirm that the naturally occurring ^176^Lu (2.59% abundance, σ_th_ = 1967 b) in the nanoparticles successfully captured neutrons and was converted into radioactive ^177^Lu via the (n,γ) reaction. However, it should be noted that the synthesized UCNPs contain multiple lanthanide elements, including ^176^Lu, ^175^Lu, ^176^Yb, and ^169^Tm. Therefore, in addition to the activation of naturally occurring ^176^Lu to ^177^Lu, neutron irradiation may also activate other stable isotopes and generate radioactive byproducts that contribute to minor background signals in the γ-ray spectrum.

For example, ^175^Lu (97.41% abundance, σ_th_ = 22 b) may be activated to metastable ^176m^Lu (T_1/2_ = 3.7 h), while ^176^Yb and ^169^Tm (100% abundance, σ_th_ = 105 b) can generate radioactive isotopes such as ^177^Yb (T_1/2_ = 1.9 h) and ^170^Tm (T_1/2_ = 128.6 days) after neutron capture. These possible byproducts should be considered when interpreting the γ-ray spectrum, especially during the early post-irradiation period. Nevertheless, the major γ-ray signals observed in this study, particularly those near 113 and 208 keV, are consistent with the characteristic γ-ray emissions of ^177^Lu, suggesting that ^177^Lu was the predominant radionuclide produced by neutron activation. Moreover, short-lived activated isotopes such as ^176m^Lu and ^177^Yb are expected to decay rapidly over hours, thereby reducing their contribution to overall radioactivity in subsequent *in vitro* and *in vivo* experiments. Therefore, although minor activation products cannot be completely excluded, the γ-ray spectral features support the successful generation of ^177^Lu from Lu-containing UCNPs.

To further confirm the radioactive decay behavior of the activated UCNPs, the quantitatively evaluate (Fig. 3b) and the radioactive decay profiles of neutron-activated NaLuF_4_:Yb,Tm@NaLuF_4_ and undoped NaLuF_4_@NaLuF_4_ were subsequently monitored, and exponential fitting yielded half-lives of 5.04 and 6.38 days, respectively (Fig. 3c). The natural logarithm of the radioactivity (ln(Activity)) was plotted against time, and the decay constant was derived using a linear regression model. The half-life of neutron-activated undoped NaLuF_4_@NaLuF_4_ was in close agreement with the theoretical half-life of ^177^Lu (6.67 days), suggesting that the discrepancy observed for NaLuF_4_:Yb,Tm@NaLuF_4_ was attributable to the incorporation of Yb and Tm dopants, thereby excluding the intrinsic decay of ^177^Lu as the primary cause of the shortened half-life. Despite this value being slightly lower than the theoretical half-life of ^177^Lu (6.67 days; Fig. 3e), this discrepancy may be attributed to self-absorption or scattering within the nanoparticle matrix, which can lead to an underestimation of gamma radiation emitted over time. Despite this minor deviation, the overall decay trend clearly indicates that the radioactivity arises from neutron-activated ^177^Lu, thereby validating the successful activation and radiolabeling of UCNPs for theranostic applications.

### 3.4 cMApt-UCNP exhibits cancer-specific permeability and therapeutic efficacy in HNSCC models

**Fig. 4:**
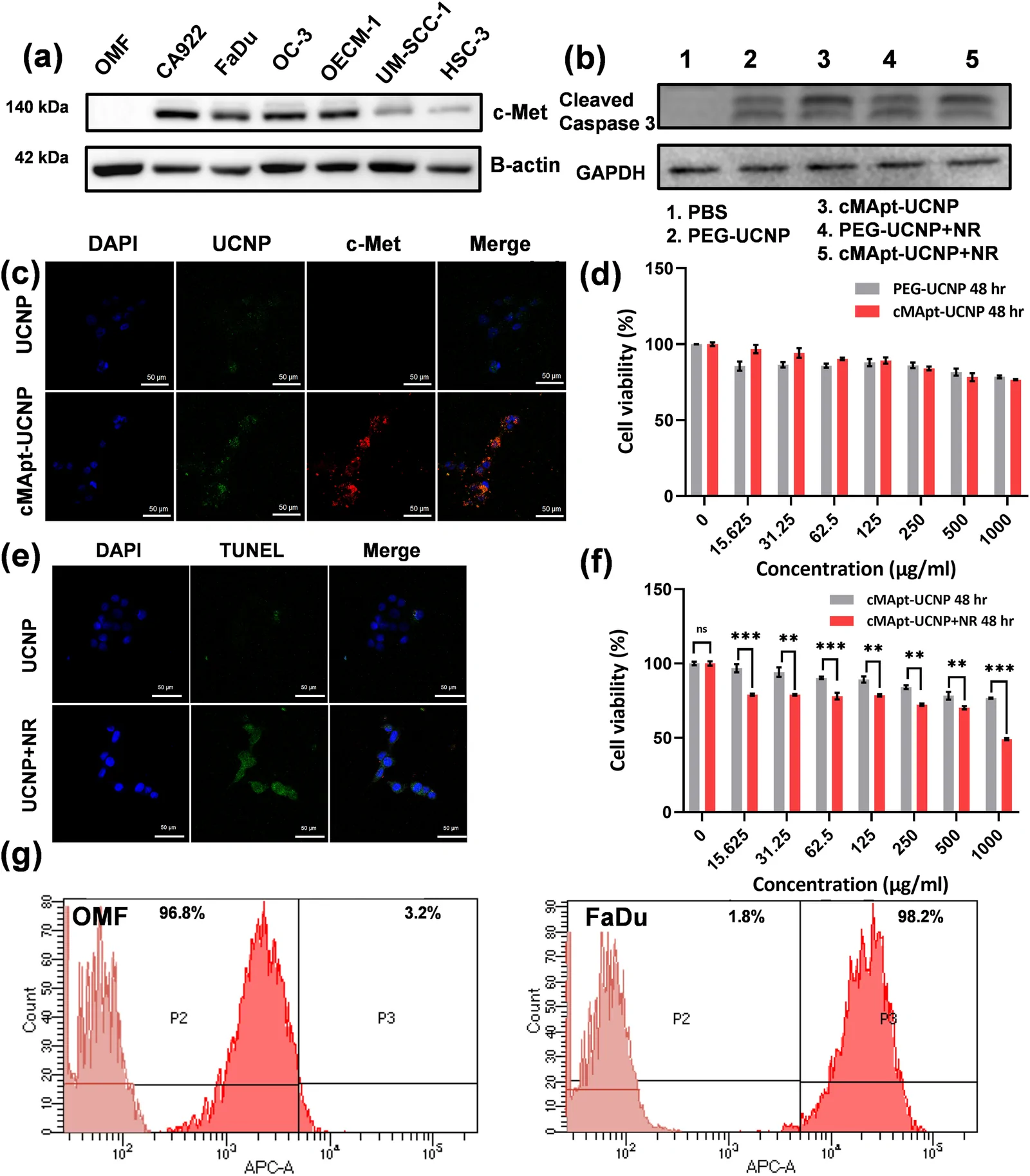
***In vitro* evaluation of UCNPs.** (a) Western blot analysis showing c-Met expression in individual HNSCC cells. The c-Met protein band was indicated at 140 kDa, confirming the presence of the target receptor. (b) Cleaved Caspase-3 expression in cells after incubation with PBS/PEG-UCNP/cMApt-UCNP/PEG-UCNP + NR/cMApt-UCNP + NR for 48 h, indicating induction of apoptosis. (c) Confocal microscopy images showing cellular uptake of PEG-UCNPs and cMApt-UCNPs (100 μg/mL) after 12 h of incubation in FaDu cells. Scale bar: 50 μm; cMApt-UCNPs show enhanced internalization. (d) Cell viability of FaDu cells after 48 h of incubation with PEG-UCNPs and Apt-UCNPs, indicating low cytotoxicity of PEG-modified nanoparticles. (e) Confocal images of TUNEL staining for PBS control and UCNP + NR (100 μg/mL, 100 μCi) after 9 h of incubation in FaDu cells. Scale bar: 50 μm; UCNP + NR induces apoptosis. (f) Cell viability of FaDu cells after 48 h of incubation with cMApt-UCNPs and cMApt-UCNPs + NR (inset: Alamar Blue color change), showing enhanced cytotoxic effect with NR loading. (g) Flow cytometry analysis of targeting efficiency of Apt-UCNPs in OMF and FaDu cells after 9 h of incubation, demonstrating specific targeting of FaDu cells. All experiments were performed with n = 5. Statistical significance was analyzed using one-way ANOVA with Tukey's post hoc test (*p < 0.05, **p < 0.01, ***p < 0.001). (For interpretation of the references to color in this figure legend, the reader is referred to the Web version of this article.)

To identify HNSCC cell lines with elevated c-Met expression, Western blot analysis was performed on one normal fibroblast OMF and six HNSCC cell lines: CA922, FaDu, OC-3, OECM-1, UM-SCC-1, and HSC-3. As shown in Fig. 4a, the expression of c-Met protein (140 kDa) varied among the cell lines tested, with β-actin (42 kDa) used as an internal loading control. Quantification of c-Met Expression in Fig. S5c shows that CA922, FaDu, and OC-3 exhibit relatively high levels of c-Met protein compared to the other cell lines. In contrast, UM-SCC-1 and HSC-3 showed significantly lower c-Met expression, whereas OECM-1 displayed moderate but variable levels. Based on these results, CA922, FaDu, and OC-3 were identified as suitable candidates for c-Met target studies. Among them, FaDu was selected for subsequent experiments due to its widespread use in cancer research and its suitability for establishing tumor xenograft models.

**Fig. 5:**
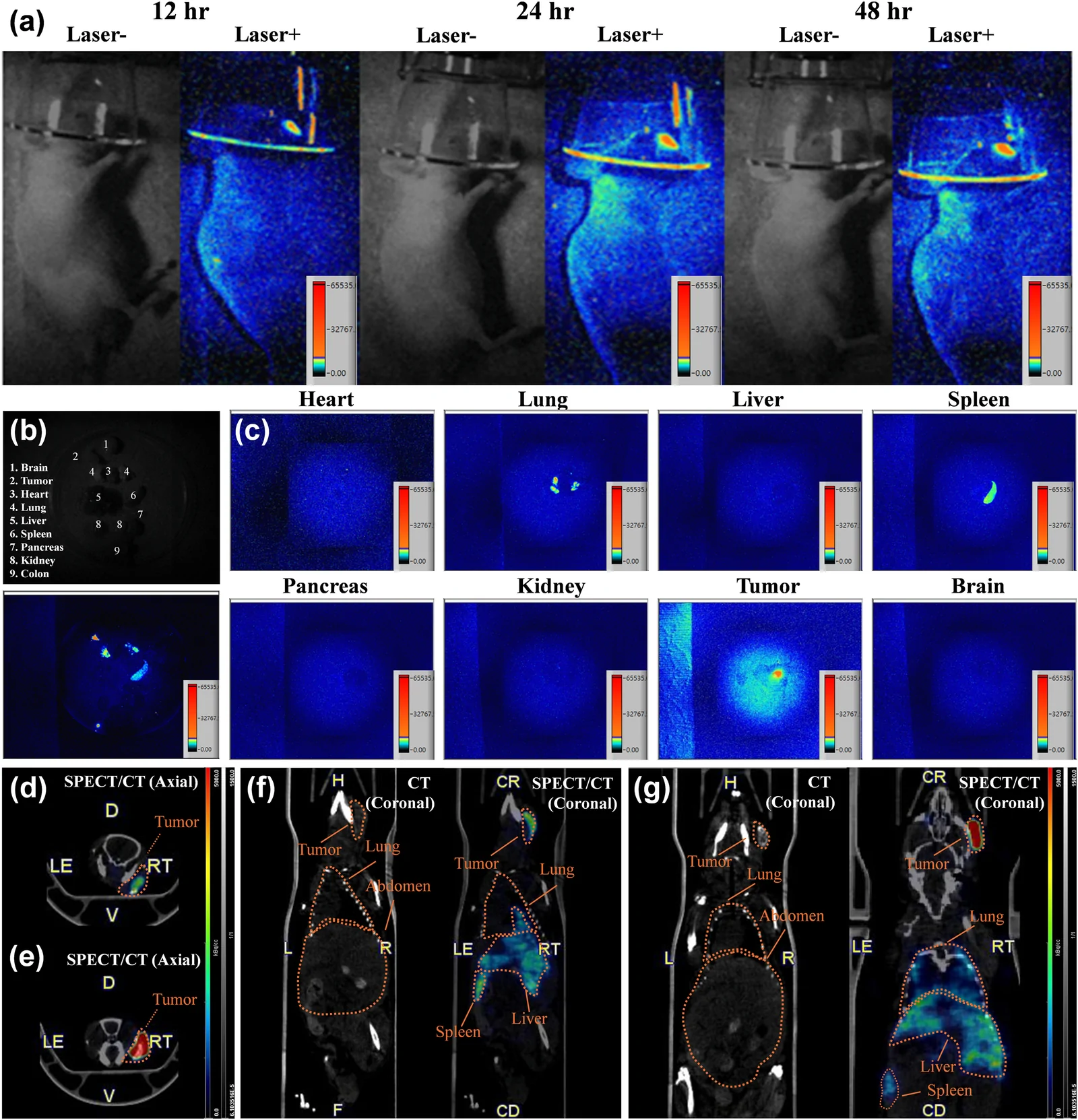
***In vivo* imaging evaluation of cMApt-UCNPs.** (a) Tumor uptake: I.V. injection of cMApt-UCNPs (10 mg/mL, 100 μL) into mice, followed by 980 nm laser excitation (20 W, 80%) at 12–48 h to observe upconversion fluorescence in the tumor region, showing strong tumor-specific signals. (b) Group overview of *ex vivo* organ imaging after 48 h. (c) Individual *ex vivo* upconversion images of heart, lung, liver, spleen, pancreas, kidney, tumor, and brain after 48 h, showing the highest fluorescence in the tumor. Axial brain SPECT/CT images show tracer uptake in cerebral and tumor regions, while whole-body SPECT/CT illustrates systemic biodistribution (inset: HNSCC tumor, lung, and liver) and anatomical localization after treating with radioactive (d, f) PEG-UCNPs and (e, g) cMApt-UCNPs.

To evaluate the cellular uptake efficiency of the synthesized nanoparticles, PEG-UCNPs and cMApt-UCNPs were added to the culture medium of FaDu cells at a concentration of 100 μg/mL. After incubation, observe the cells under a light microscope. As shown in Fig. S6, cells treated with PEG-UCNPs exhibited a limited degree of nanoparticle internalization. In contrast, cells treated with cMApt-UCNPs showed a significant accumulation of nanoparticles, resulting in visible black masses. This enhanced uptake was attributed to the specific binding of the c-Met aptamer on the nanoparticle surface to the overexpressed c-Met receptor on FaDu cells, thereby promoting receptor-mediated endocytosis. To further verify the targeted uptake of the synthesized nanoparticles, confocal laser scanning microscopy was performed on FaDu cells treated with unmodified UCNPs or cMApt-UCNPs at a concentration of 100 μg/mL. UCNPs exhibit intrinsic green upconversion fluorescence under 980 nm laser excitation, while the c-Met aptamer on cMApt-UCNPs was labeled with Cy5 (red fluorescence; excitation: 649 nm, emission: 670 nm). As shown in Fig. 4c, cells treated with unmodified UCNPs exhibited minimal green fluorescence (Excitation at 980 nm; Emission peaks at 475/650/700 nm) and no detectable red signal, indicating limited internalization and lack of targeting ability. In contrast, cells incubated with cMApt-UCNPs exhibited strong colocalized green and red fluorescence, confirming the presence of UCNPs and Cy5-labeled c-Met aptamers within the cells. The merged images demonstrated substantial overlap between the UCNP and Cy5-labeled c-Met signals, indicating successful receptor-mediated internalization of cMApt-UCNPs through the c-Met pathway. Three-dimensional (3D) confocal reconstruction (Fig. S7a and S7b) confirmed the intracellular localization of cMApt-UCNPs, providing direct evidence of efficient nanoparticle uptake. To evaluate the targeting specificity of cMApt-UCNPs toward c-Met-expressing cells, flow cytometry was performed on OMF cells, which express low levels of c-Met, and FaDu cells, which express high levels of c-Met. Cells were incubated with 500 μg/mL Cy5-labeled cMApt-UCNPs for 9 h. As shown in Fig. 4g, only 3.2% of OMF cells were Cy5-positive (P3 region), indicating minimal nanoparticle binding and uptake. In contrast, 98.2% of FaDu cells exhibited strong Cy5 fluorescence, demonstrating efficient binding and internalization of cMApt-UCNPs in c-Met-overexpressing cells.

To investigate whether the c-Met aptamer retained its function after conjugation to UCNPs, FaDu cells were treated with 500 μg/mL of PEG-UCNPs or cMApt-UCNPs for 24, 48, and 72 h. As shown in Fig. S5a and further quantified in Fig. S5b, treatment with cMApt-UCNPs led to a time-dependent decrease in c-Met protein expression. Specifically, c-Met expression was reduced to 56% of the control level at 24 h, remained suppressed at approximately 68% at 48 h, and further decreased to 25% at 72 h. These results indicate that the c-Met aptamer retains its bioactivity after binding to nanoparticles, effectively downregulating c-Met expression over time. This suggests that cMApt-UCNPs can not only serve as target carriers but also contribute to therapeutic inhibition of the c-Met pathway. Interestingly, PEG-UCNPs also resulted in a slight decrease in c-Met expression over time. This may be attributed to nonspecific nanoparticle-induced cellular stress indirectly inhibiting the expression of specific growth factor receptors, as nanomaterials have been reported to affect downstream signaling pathways, such as MAPK and PI3K/Akt, which are closely related to c-Met regulation. But further research is needed to elucidate the exact mechanism behind this observation.

To further confirm that nanoparticle uptake was mediated by specific c-Met receptor recognition rather than nonspecific cellular association, competitive inhibition assays were performed using a free c-Met aptamer. FaDu cells were pre-treated with free and pure c-Met aptamer (100 or 300 nmol) for 4 h prior to incubation with Cy5.5-labeled cMApt-UCNPs. As shown in Fig. S8, confocal microscopy revealed a marked reduction in both green upconversion fluorescence and Cy5 fluorescence following pre-treatment with free aptamer compared with the untreated control, indicating that occupation of c-Met receptors effectively inhibited the binding and internalization of cMApt-UCNPs. Consistent with the confocal observations, flow cytometric analysis demonstrated a concentration-dependent reduction in nanoparticle uptake after receptor blocking (Fig. S9a–S9c). The Cy5-positive cell population decreased from 86.3% in the control group to 69.2% and 65.7% following pre-treatment with 100 and 300 nmol free c-Met aptamers, respectively, while the Cy5-negative population increased correspondingly from 13.7% to 30.8% and 34.3%. These findings demonstrate that free c-Met aptamer competitively blocked the interaction between cMApt-UCNPs and cellular c-Met receptors, thereby significantly reducing nanoparticle internalization. Collectively, confocal imaging, flow cytometric analysis, and competitive inhibition assays consistently demonstrate that cMApt-UCNPs specifically recognize and bind to c-Met-overexpressing cells through receptor-mediated targeting, providing strong evidence for the targeting specificity of the c-Met aptamer-functionalized UCNP platform.

To evaluate the biocompatibility of the synthesized nanoparticles, cytocompatibility assays were first conducted using normal oral mucosal fibroblasts (OMF) and FaDu head and neck squamous cell carcinoma cells. As shown in Fig. S10a–S10d, both PEG-UCNPs and cMApt-UCNPs exhibited excellent biocompatibility in OMF cells, with cell viability remaining consistently above 80% after 24 and 48 h of incubation across all tested concentrations. These results indicate minimal cytotoxicity toward normal cells, confirming the high biosafety of the surface-functionalized UCNPs. Similarly, cytocompatibility was evaluated in FaDu cancer cells to exclude intrinsic nanoparticle-induced toxicity prior to therapeutic studies. As shown in Fig. 4d–S11a, and S11b, treatment with PEG-UCNPs or cMApt-UCNPs alone resulted in cell viability exceeding 80% at both 24 and 48 h, even at high concentrations, demonstrating that neither PEGylation nor c-Met aptamer modification induces appreciable cytotoxicity in the absence of neutron activation. To assess therapeutic efficacy, FaDu cells were treated with neutron-activated cMApt-UCNPs (cMApt-UCNP + NR). In contrast to the non-activated controls, Fig. 4f shows a pronounced and dose-dependent reduction in cell viability following neutron irradiation. Cell viability decreased to approximately 50% at higher nanoparticle concentrations, indicating significant cytotoxic effects. This marked difference between activated and non-activated groups confirms that the observed cell killing arises from neutron-induced activation of the lutetium-containing UCNPs, consistent with β-particle–mediated damage resulting from the decay of ^177^Lu. Overall, these results demonstrate that both PEG-UCNPs and cMApt-UCNPs possess excellent biocompatibility toward normal and cancer cells prior to activation, while neutron-activated cMApt-UCNPs exhibit potent and selective therapeutic cytotoxicity, supporting their suitability for neutron-activated radiotherapeutic applications.

To evaluate the apoptotic effect induced by neutron-activated nanoparticles, FaDu cells treated with UCNP + NR (500 μg/mL, 100 μCi) for 9 h were subjected to TUNEL (terminal deoxynucleotidyl transferase dUTP nick end labeling) staining. Nuclear morphology (DAPI, blue) and DNA fragmentation (TUNEL, green) were observed using confocal laser scanning microscopy. As shown in Fig. 4e, cells in the control group (PBS-treated) exhibited intact nuclei and minimal TUNEL signals, indicating negligible apoptosis. In contrast, cells treated with UCNP + NR showed strong green fluorescence localized in the nucleus, indicating extensive DNA fragmentation—a hallmark of apoptosis. The merged images confirmed the colocalization of TUNEL signals with the nuclear region, indicating that neutron-activated UCNPs could effectively induce apoptosis via DNA damage. To explore whether neutron-activated cMApt-UCNPs induce apoptosis via activated caspase-3, we performed Western blot analysis to detect total and cleaved caspase-3 in FaDu cells treated with cMApt-UCNP + NR for 6 and 24 h. GAPDH was used as an internal control. As shown in Fig. 4b and further quantified in Fig. S5d, treatment with cMApt-UCNP + NR resulted in a significant, time-dependent increase in cleaved caspase-3 expression. Compared with the untreated control group, cells treated for 6 h already exhibited a clear elevation in cleaved caspase-3, which became more pronounced at 24 h, indicating progressive activation of apoptosis. Quantification of band intensity confirmed that β^−^ emission from neutron-activated UCNPs effectively induced apoptosis through caspase-3 activation. To further verify the therapeutic effect of the material, a colony formation assay was performed on FaDu cells (Fig. S12). PEG-UCNPs and cMApt-UCNPs alone exhibited negligible cytotoxicity, with colony numbers comparable to the control group. In contrast, neutron-activated UCNPs significantly reduced the number of colonies, among which cMApt-UCNPs + NR showed the most potent inhibitory effect. Quantitative analysis revealed that the average colony number in the cMApt-UCNP + NR group was decreased by more than 80% compared with the untreated control group, indicating impaired proliferative potential. These findings demonstrate that cMApt-UCNPs, upon neutron irradiation, induce sustained cytotoxicity sufficient to inhibit long-term clonal survival of HNSCC cells.

### 3.5 In vivo evaluation of tumor targeting imaging and therapeutic efficacy

**Fig. 6:**
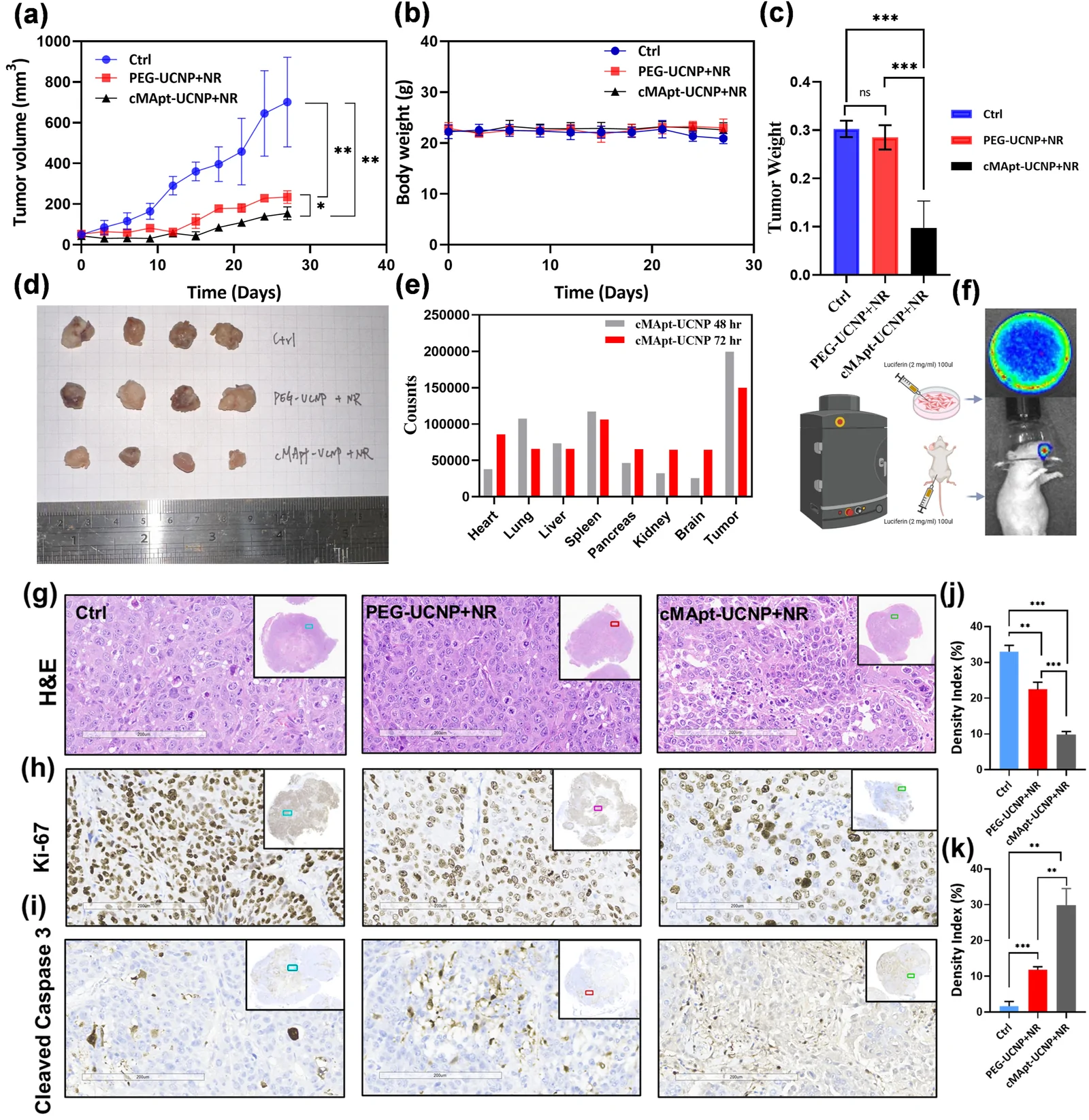
***In vivo*, histological and immunohistochemical evaluation of tumor sections after treatment.** (a) Tumor volume was measured with a vernier caliper at different time points, indicating effective tumor growth inhibition by cMApt-UCNP + NR treatment. (b) Body weight of mice after treatment, showing no significant systemic toxicity. (c) Tumor weight of mice after treatment, demonstrating a significant reduction compared to controls. (d) *Ex vivo* photographs of excised solid tumors, visually confirming tumor suppression. (e) Quantitative analysis of *ex vivo* fluorescence intensity in different organs. (f) The IVIS detection of cells infected with luciferin produced bioluminescent signals, which were subsequently used for localization and confirmation. (g) H&E staining of tumor tissues from control, PEG-UCNP + NR, and cMApt-UCNP + NR groups. The cMApt-UCNP + NR group exhibited the most pronounced reduction in tumor cellularity and nuclear integrity. (h) Ki-67 IHC staining indicated markedly decreased proliferative activity in the cMApt-UCNP + NR group relative to the control and PEG-UCNP + NR groups (scale bar: inset, 2 mm; enlarged, 200 μm). (i) Cleaved caspase-3 staining revealed enhanced apoptotic activity in the cMApt-UCNP + NR group. Quantification of (j) Ki-67 and (k) cleaved caspase-3 confirmed significant inhibition of proliferation and increased apoptosis upon cMApt-UCNP + NR treatment. All experiments were performed with n = 5. Statistical significance was analyzed using Two-way ANOVA (*p < 0.05, **p < 0.01, ***p < 0.001).

To establish an orthotopic HNSCC model, 2 × 10^5^ FaDu-GL cells (expressing luciferase) were injected into the right buccal region of nude mice. One week after tumor implantation, *in vivo* bioluminescence imaging was performed using an IVIS system to monitor tumor formation and luciferase activity. The overall experimental procedure, including tumor implantation, monitoring, imaging, and subsequent treatment, is illustrated in the flow chart (Fig. S13). As shown in Fig. 6f, a strong luminescent signal was detected at the tumor site, confirming that FaDu-GL cells were successfully implanted and grown in the Head and Neck region. Consistent and localized bioluminescence enables subsequent experiments to monitor tumor progression and treatment response longitudinally. To verify the *in vivo* tumor-targeting ability and biodistribution of cMApt-UCNPs, tumor-bearing mice were intravenously injected with nanoparticles at a concentration of 10 mg/mL (equivalent to 40 mg/kg body weight). Upconversion fluorescence imaging was conducted using a 980 nm near-infrared laser (20 W) to excite the UCNPs at 12, 24, and 48 h post-injection. As shown in Fig. 5a, strong fluorescent signals were detected at the tumor site under laser excitation at all time points, indicating efficient tumor accumulation and sustained retention of cMApt-UCNPs. These findings highlight the potential of cMApt-UCNPs for real-time *in vivo* imaging and tracking. Since the goal of this study was to apply neutron-activated cMApt-UCNPs (cMApt-UCNPs + NR) for targeted radiotherapy, this non-radioactive imaging experiment was performed in advance to simulate the biodistribution of the therapeutic formulation. Sufficient tumor accumulation is essential to ensure that, upon neutron activation, the radioactive nanoparticles localize at the tumor site and effectively inhibit tumor growth via β-particle emission. To further evaluate biodistribution, major organs, including the heart, lungs, liver, spleen, pancreas, kidneys, brain, and tumor, were harvested for 48 h post-injection for *ex vivo* fluorescence imaging. As shown in Fig. 5b and c, and video S1, the strongest fluorescence signal was observed in the tumor, with only mild to moderate signals in the lungs and spleen, and minimal distribution to other organs. This confirms the tumor-targeting capability of cMApt-UCNPs and supports their suitability for systemic delivery in targeted radiotherapeutic applications. Quantitative analysis of fluorescence intensity in different organs (Fig. 6e) reveals that the tumor signal was approximately 3.8-fold higher than in the liver, 5.2-fold higher than in the lungs, and 6.7-fold higher than in the kidneys, supporting the suitability of cMApt-UCNPs for systemic delivery in targeted radiotherapeutic applications.

To assess the tumor-targeting capability of cMApt-UCNPs, *in vivo* fluorescence imaging was conducted in tumor-bearing mice following intravenous injection of either PEG-UCNPs (non-targeted) or cMApt-UCNPs (targeted). Imaging was performed at 24, 48, and 72 h post-injection using 980 nm laser excitation to induce upconversion luminescence. As shown in Fig. S14, non-laser (Laser–) images served as background controls, while laser-activated (Laser+) images revealed distinct upconversion emission signals. In the PEG-UCNP group, only weak fluorescence was observed at the tumor site (indicated by red circles) across all time points, suggesting limited tumor accumulation due to the absence of active targeting.

In contrast, the cMApt-UCNP group exhibited markedly stronger fluorescence localized at the tumor site, with the most intense signal observed at 48 h post-injection, indicating peak nanoparticle accumulation. These findings confirm that conjugation with the c-Met aptamer significantly enhances the tumor-targeting efficiency of UCNPs via active recognition mechanisms. Moreover, the temporal fluorescence profiles of the head and neck suggest that 48 h post-injection represents the optimal window for imaging or therapeutic application of this targeted nanoplatform. In addition to optical imaging, we employed the radioisotope Lutetium-177 (^177^Lu) as a SPECT/CT contrast/theranostic agent [38]. Compared with conventional ^177^Lu labeling approaches that rely on surface chelation or ligand conjugation, our neutron-activated NaLuF_4_-based nanoparticles provide a fundamentally more stable and reliable theranostic platform. The UCNP platform used had a NaLuF_4_ host lattice; the Lu component was neutron-activated to generate ^177^Lu, enabling both therapeutic and imaging functions to be performed simultaneously. Because ^177^Lu emits β-particles for therapy and γ-rays (∼113 keV and 208 keV) for SPECT imaging, a single material can perform tumor irradiation while enabling SPECT quantification, thus achieving true theranostics. Indeed, quantitative SPECT with ^177^Lu has been demonstrated to be feasible in small-animal systems and clinical settings. The NaLuF_4_ host lattice offers intrinsic advantages: the high atomic number of lutetium provides strong X-ray attenuation (beneficial for CT) and supports the incorporation of activated Lu isotopes within the crystal lattice (minimizing leaching and improving *in vivo* retention). Previously, NaLuF_4_ UCNPs were studied for multimodal CT/MR/UCL imaging owing to their heavy-element Lu core. In our study, the neutron-activated UCNPs therefore provide a unified platform: optical upconversion luminescence, CT contrast, and SPECT via ^177^Lu gamma emission. Following surface modification of the particles with cMApt, the cMApt-UCNPs exhibited significantly enhanced accumulation in FaDu tumor tissue at the 16-h post-injection time point (500 μCi, 200 μL injection) compared with PEG-UCNPs. While some off-target accumulation remained (notably in lung and liver), the tumor-to-background ratio was markedly improved with the c-Met aptamer ligand. In the corresponding SPECT/CT images, the targeted group showed distinctly higher uptake in the tumor region in both axial and coronal planes (see Fig. 5d and f for PEG-UCNPs, Fig. 5e and g for cMApt-UCNPs; Supplementary Fig. S15a and S15b shows the axial vs coronal comparison).

Quantitative image analysis of the NanoSPECT/CT Mediso acquisitions demonstrated that tumor uptake of the cMApt-UCNPs was significantly elevated compared with the non-targeted PEG group. As summarized in Table S2, the cMApt-UCNP + NR group exhibited a tumor radioactivity of 11,928 ± 9235 kBq/cc, which was substantially higher than that of the PEG-UCNP + NR group (1102 ± 168 kBq/cc). Correspondingly, the tumor-to-spleen (T/S) and tumor-to-liver (T/L) ratios increased from 1.012 to 0.98 to 13.87 and 10.95, respectively, demonstrating a substantially enhanced tumor-to-background contrast and selective tumor retention after c-Met aptamer modification. The high spatial-resolution SPECT/CT images enabled co-registration of the functional SPECT signal with the anatomical CT, allowing precise localization of the tracer in the tumor, liver, and lung. The difference between axial and coronal views underlines the importance of multi-plane imaging for accurate biodistribution assessment. Prior work has shown that variability between systems and reconstruction methods must be accounted for when performing ^177^Lu SPECT quantification. Our data support the feasibility of such theranostic nano-platforms, although optimization of particle pharmacokinetics (reducing lung/liver accumulation and increasing tumor retention) remains necessary. The dual-modal function of our ^177^Lu-activated NaLuF_4_ UCNPs for imaging and therapy, together with aptamer-based tumor targeting, has been validated *in vivo* using SPECT/CT. Taken together, this intrinsic ^177^Lu activation strategy overcomes the major limitations of surface-labeled radiotracers, offering superior *in vivo* stability, effective tumor localization, and minimized risk of off-target irradiation. Dual-plane imaging (optical and SPECT/CT) confirmed tumor uptake and overall biodistribution patterns, supporting the feasibility of this theranostic strategy.

However, we are concerned that a more detailed evaluation of the longitudinal *in vivo* behavior, including circulation dynamics and clearance characteristics, is important for a better understanding of the translational potential of this nanoplatform. To address this concern, we performed additional longitudinal *in vivo* tracking studies using SPECT/CT imaging up to 48 h post-injection, together with gamma-counter analyses of blood samples collected at 72, 96, and 120 h post-injection. The longitudinal SPECT images (Fig. S16a–S16c, and video S2) demonstrated a clear time-dependent redistribution of radioactivity. At early time points, radioactivity was broadly distributed throughout the body, whereas by 48 h post-injection, the signal was predominantly confined to the liver, with minimal retention observed in other tissues. These findings suggest progressive clearance of the nanoparticles from systemic circulation, accompanied by transient hepatic retention, consistent with the expected *in vivo* behavior of nanoparticles with a hydrodynamic diameter of approximately 200 nm.

In parallel, blood gamma-counter analyses revealed consistently low levels of residual radioactivity at all examined time points. The corresponding blood CPM, %ID, and %ID/g values are summarized in Table S3. These findings indicate minimal residual circulation of the neutron-activated nanoparticles and suggest that most nanoparticles had been cleared from the bloodstream by 72 h post-injection, thereby minimizing prolonged systemic radiation exposure. In addition, no significant changes in body weight or observable signs of systemic toxicity were detected during the experimental period. Collectively, these additional studies provide important insights into the biodistribution, circulation behavior, clearance profile, and *in vivo* retention of the developed nanoplatform, thereby strengthening its potential applicability for multimodal radionuclide theranostics.

To evaluate the therapeutic effect of neutron-activated cMApt-UCNPs (cMApt-UCNP + NR), tumor-bearing mice were treated, and tumor progression was monitored over a 27-day period. As shown in Fig. 6a, tumor volumes in the control group increased progressively throughout the study, whereas PEG-UCNP + NR treatment resulted in only a moderate reduction in tumor growth. In contrast, mice treated with cMApt-UCNP + NR exhibited pronounced and sustained inhibition of tumor progression, demonstrating a potent anti-tumor effect. To further assess the consistency and robustness of the therapeutic response, individual tumor growth curves for each mouse are presented in Fig. S17. Each line represents tumor volume progression in a single animal over the treatment period. Tumors in the control and PEG-UCNP + NR groups exhibited heterogeneous yet continuous growth, whereas mice treated with cMApt-UCNP + NR showed consistently suppressed tumor progression across all individuals, indicating a reproducible, reliable therapeutic outcome rather than an effect driven by outliers. Throughout the treatment period, the body weights of mice in all groups remained relatively stable (Fig. 6b), indicating no apparent systemic toxicity associated with the treatments. At the end of the study, tumors were excised for direct visual comparison (Fig. 6d). Tumors from the cMApt-UCNP + NR group were markedly smaller than those from the control and PEG-UCNP + NR groups.

Quantitative analysis of tumor weight (Fig. 6c) further confirmed these observations: the mean tumor weight in the cMApt-UCNP + NR group was significantly lower than in both the control and PEG-UCNP + NR groups, whereas no significant difference was observed between the PEG-UCNP + NR and control groups. Collectively, these results demonstrate that cMApt-UCNPs exert a strong therapeutic effect *in vivo* following neutron activation. The enhanced tumor inhibition is attributed to the dual functionality of this system, which combines selective tumor accumulation via c-Met aptamer targeting with localized β-particle irradiation from neutron-activated ^177^Lu decay. Importantly, the stable body weight profiles further indicate favorable *in vivo* safety, supporting neutron-activated cMApt-UCNPs as a safe and effective strategy for targeted cancer therapy. Histopathological and immunohistochemical analyses were performed to evaluate the therapeutic outcomes of various treatments in FaDu cell xenograft tumors. As shown in Fig. 6g, hematoxylin and eosin (H&E) staining of tumor sections showed that the control group exhibited densely packed tumor cells with preserved nuclear morphology, consistent with active proliferation. Tumors treated with PEG-UCNP + NR displayed partial changes, including a modest reduction in cellular density and nuclear integrity. In comparison, the cMApt-UCNP + NR group showed more pronounced alterations, including reduced cellular density, atomic shrinkage and fragmentation, and regions suggestive of cell clearance, indicating a more substantial inhibitory effect on tumor progression. To further probe the biological effects, immunohistochemistry (IHC) was performed using Ki-67 as a proliferation marker and cleaved caspase-3 as an apoptotic marker. As shown in Fig. 6h, Ki-67 expression was strongly detected in the control group, indicating high proliferative activity, whereas treatment with PEG-UCNP + NR led to a moderate reduction. Notably, the cMApt-UCNP + NR group showed markedly weaker Ki-67 staining, indicating significant suppression of proliferative capacity. Conversely, cleaved caspase-3 staining (Fig. 6i) revealed a distinct pattern: the control group showed minimal apoptotic activity, and PEG-UCNP + NR induced only a mild increase, while the cMApt-UCNP + NR group exhibited robust and widespread cleaved caspase-3 positivity, highlighting the induction of apoptosis. Quantitative analyses (Fig. 6j and k) corroborated these findings, demonstrating significantly reduced Ki-67 indices and elevated cleaved caspase-3 levels in the cMApt-UCNP + NR group compared to the other groups. Collectively, these results provide strong evidence that cMApt-UCNPs, in combination with neutron radiation, exhibit potent antitumor efficacy by simultaneously inhibiting proliferation and facilitating apoptosis. Furthermore, H&E staining of major organs was performed to assess potential systemic toxicity after treatment. As shown in Fig. S18, the control, PEG-UCNP + NR, and cMApt-UCNP + NR groups showed generally preserved tissue morphology, with no obvious signs of tissue damage, inflammation, or necrosis. These findings suggest that the treatments did not cause evident histological toxicity under experimental conditions.

## 4 Conclusion

This study reports the successful development of a multifunctional theranostic nanoplatform by synthesizing c-Met aptamer-conjugated upconversion nanoparticles (cMApt-UCNPs) for targeted imaging and neutron-capture-based radiotherapy with lutetium-177 (Lu-177) in HNSCC. The cMApt-UCNPs exhibited high tumor-targeting specificity by selectively binding to c-Met-overexpressing cancer cells. *In vitro* studies confirmed that the nanoparticles effectively downregulated c-Met protein expression, induced apoptosis, and suppressed colony formation in HNSCC cells. Upon neutron activation, Lu-176 embedded in the nanoparticles was efficiently converted to Lu-177, a therapeutic radioisotope that emits β-particles to induce DNA damage and inhibit tumor proliferation. Beyond serving as an optical tracking component, the upconversion luminescence capability enables sensitive, longitudinal visualization of nanoparticle localization with high spatial resolution, whereas the intrinsic radiochemical properties of Lu-177 provide quantitative SPECT imaging to evaluate radionuclide distribution and therapeutic radiation delivery. In addition, the platform enabled complementary dual-modality imaging using near-infrared (NIR) fluorescence and SPECT, where optical imaging facilitated non-invasive monitoring of nanoparticle trafficking and tumor accumulation, while SPECT provided radionuclide-specific biodistribution analysis and dosimetric assessment. *In vivo* studies further validated the enhanced tumor localization and potent antitumor efficacy of cMApt-UCNPs, demonstrating significant inhibition of tumor growth with minimal off-target effects. By integrating complementary imaging capabilities with radionuclide therapy within a single nanoparticle platform, this strategy offers a promising advance in the personalized treatment of HNSCC, improving therapeutic specificity, reducing systemic toxicity, and enabling longitudinal monitoring of nanoparticle behavior and therapeutic response.

## CRediT authorship contribution statement

**Kai-Hung Lin:** Writing – original draft, Investigation, Formal analysis, Data curation. **Chun-Yi Wu:** Writing – original draft, Validation, Resources, Methodology. **Yu-Chan Chang:** Writing – original draft, Validation, Resources, Investigation. **Ming-Hsien Chan:** Writing – review & editing, Writing – original draft, Supervision, Funding acquisition, Conceptualization.

## Declaration of competing interest

The authors declare that they have no known competing financial interests or personal relationships that could have appeared to influence the work reported in this paper.

## Data Availability

Data will be made available on request.

## Abbreviations

NIR: Near Infrared Light
SPECT: Single Photon Emission Computed Tomography
PEG did-acid 3000: Polyethylene Glycol 3000
c-Met: Mesenchymal-Epithelial Transition Factor Receptor
Lu: Lutetium-177
UCNP: Upconversion Nanoparticle
PEG-UCNP: Polyethylene Glycol 3000-modified Upconversion Nanoparticle
cMApt-UCNP: c-Met Aptamer-modified Polyethylene Glycol 3000 Upconversion Nanoparticle

## Appendix A Supplementary data

The Supporting Information contains full experimental procedures and characterization details used in this study, including an instrument list (XRD, TEM, EDX, DLS/zeta, FTIR, PL, and UV–Vis), expanded methods for NIR-II imaging, IVIS, cell viability and toxicity assays, animal protocols, and tissue staining (H&E and IHC). The following files are available free of charge.
