# Supplementary material for: Upconversion nanoplatform unlocks the theranostic potential of Lu-177 radiotherapy via c-MET axis targeting in head and neck squamous cell carcinoma
Source: Mater Today Bio. 2026 Aug 25;40:103585. doi: 10.1016/j.mtbio.2026.103585 (PMC13545463; doi:10.1016/j.mtbio.2026.103585)
Supplement: Supplementary file 1 [file mmc1.docx]

Supporting Information

Upconversion Nanoplatform Unlocks the Theranostic Potential of Lu-177 Radiotherapy via c-MET Axis Targeting in Head and Neck Squamous Cell Carcinoma

*Kai-Hung Lin ^a^, Chun-Yi Wu ^a,^*, Yu-Chan Chang ^a,^*, and Ming-Hsien Chan ^a,^**

^a^ Department of Biomedical Imaging and Radiological Sciences, National Yang Ming Chiao Tung University, Taipei 112, Taiwan.

*Corresponding Authors’ E-mail addresses:*

[chunyiwu@nycu.edu.tw](mailto:chunyiwu@nycu.edu.tw) (C. Y. Wu)

[yuchanchang@nycu.edu.tw](mailto:yuchanchang@nycu.edu.tw) (Y. C. Chang)

[mhchan@nycu.edu.tw](mailto:mhchan@nycu.edu.tw) (M. H. Chan)

**Contents**

1. Characterizations	2
2. Experimental procedures	4
   2.1 – 2.7 Analytical instruments	4
   2.8 Near-infrared (NIR)-II imaging	.5
   2.9 Cell viability and toxicity tests	5
   2.10 Animal studies	5
   2.11 *In Vivo* imaging system; IVIS	5
   2.12 Tissue staining assay	6
3. Supplementary figures	7
4. Supplementary tables	21

**1. Characterizations:**

| **Instrument Name** | **Instrument brand** | **Instrument location** |
| --- | --- | --- |
| X-ray diffractometer (XRD) | Bruker D8 Advance Plus | Academia Sinica Applied Science and Technology Center, 6th Floor, Mu-Huai Laboratory |
| Transmission Electron Microscopy (TEM) | JEOL JEM-1400plus | Room B104, 2F, Experimental Building, Yang Ming Campus |
| Energy Dispersive X-Ray Microanalysis (EDX) | OXFORD EDS X-Max 80T | Room B104, 2F, Experimental Building, Yang Ming Campus |
| Dynamic Light Scattering (DLS) | Nano-ZS | Department of Chemical Engineering, National Taiwan University, Powder and Particle Laboratory, Chemical Engineering Building 1, Room 107 |
| Zeta potential | Nano-ZS | Department of Chemical Engineering, National Taiwan University, Powder and Particle Laboratory, Chemical Engineering Building 1, Room 107 |
| Photoluminescence (PL) | HORIBA | Academia Sinica Joint Instrument Center, 5th floor |
| Vis-NIR live real-time imaging detection system. | ISUZU OPTICS CORP | NYCU Biomedical Engineering Building Room 461/Room 462 |
| Fourier-transform infrared spectroscopy (FTIR) | Perkin Elmer Spectrum 100 | Department of Chemical Engineering, National Taiwan University, Instrumental Analysis Laboratory Room 127, Chemical Engineering Building 1 |
| Ultraviolet-visible spectroscopy (UV-Vis) | BECKMAN COULTER  DU 800 SPECTROPHOTOMETER | Yang-Ming Campus  Biomedical Engineering Building 4F. Room 465 |
| Gel Electrophoresis Analysis | ADVANCE Mupid-2plus | NYCU Biomedical Engineering Building Room 461/Room 462 |
| Ion Chamber VGH | GENIC2000 | Taipei Veterans General Hospital International Medical Service Center |
| Ion Chamber | YMRPL-D-02 | NYCU Biomedical Engineering Building Room 238 |
| Confocal | Leica TCS SP5 Confocal Spectral Microscope Imaging System | Academia Sinica, Genome Research Center, 5th Floor |
| Flow cytometry | BD FACS Canto II | Academia Sinica, Genome Research Center, 5th Floor |
| SPECT/CT | L814Nano SPECT/CT | Linkou Chang Gung Laboratory Animal Center & Molecular Translational Medicine Center |

**2. Experimental procedures (additional)**

*2.1 X-ray diffraction; XRD:* XRD was used to characterize the crystalline structure of the nanoparticles. Cu Kα radiation (λ ≈ 1.54 Å) was generated from a copper target and directed onto the samples. Diffraction patterns were collected using a θ–2θ scan, and phase identification and lattice parameters were determined by comparison with standard databases and Rietveld refinement. The results confirmed that the upconversion nanoparticles exhibited a hexagonal β-NaLuF₄ phase with appropriate dopant incorporation.

*2.2 Transmission electron microscopy; TEM:* TEM was used to characterize nanoparticle morphology and size. A high-energy electron beam (40 kV and 25 mA) was focused onto samples mounted on copper grids, and images were collected under high vacuum to ensure optimal resolution. High-resolution TEM provided detailed images of particle shape and size, and size distributions were quantified using ImageJ.

*2.3 Energy dispersive X-ray microanalysis; EDX:* EDX was performed using a system integrated with a Transmission electron microscopy (TEM) to analyze the elemental composition of the nanoparticles. Characteristic X-rays generated by electron-beam excitation provided qualitative and semi-quantitative elemental profiles. EDX mapping confirmed the spatial uniformity and successful incorporation of rare-earth dopants.

*2.4 Dynamic light scattering; DLS & Zeta potential:* DLS was used to measure the hydrodynamic diameter and polydispersity index (PDI) of the nanoparticles by analyzing their Brownian motion and applying the Stokes–Einstein equation. Measurements were performed before and after PEG and aptamer coating to monitor size changes and ensure colloidal stability. Zeta potential analysis was conducted to evaluate the surface charge and dispersion stability of the nanoparticles. An applied DC field induced electrophoretic motion, and the resulting mobility was converted to zeta potential using the Henry equation. Higher absolute values indicated stronger electrostatic repulsion and improved colloidal stability.

*2.5 Fourier transform infrared spectroscopy; FTIR:* FTIR was used to analyze functional groups of the nanoparticles. Infrared absorption spectra were recorded to identify characteristic vibrations, providing structural fingerprints. FTIR spectra before and after PEG modification confirmed successful grafting of hydrophilic PEG chains, supporting subsequent assessments of biocompatibility and functionality.

*2.6 Photoluminescence; PL:* The photoluminescence (PL) spectra of the upconversion nanoparticles (UCNPs) were recorded using a spectrometer equipped with a 980 nm laser as the excitation source. The emission signals were dispersed through a monochromator and detected with a photomultiplier tube. UCNPs dispersed in cyclohexane (UCNP, oil phase), PEG-modified UCNPs (PEG-UCNP), and c-Met aptamer-conjugated UCNPs (cMApt-PEG-UCNP) were compared to evaluate their optical properties. As shown in Figure (b), all samples exhibited characteristic emission peaks at ~475 nm (^1^G₄ → ^3^H₆), ~650 nm (^1^G₄ → ^3^F₄), and ~700 nm (^3^H₄ → ^3^H₆), with the strongest emission intensity observed for the oil-phase UCNPs, while PEG and aptamer modifications led to a slight reduction in intensity due to surface passivation and surface-state changes.

*2.7 Ultraviolet-visible spectroscopy; UV-Vis:* UV–Vis spectroscopy (200–800 nm) was used to analyze the optical properties of the nanoparticles. Spectra collected before and after surface modification revealed the characteristic absorption band of the c-Met aptamer, confirming its successful conjugation to the upconversion nanoparticles.

*2.8 Near-infrared (**NIR)-II imaging:* A small-animal *in vivo* fluorescence imaging system (visible, NIR-I, and NIR-II) equipped with a cooled InGaAs camera (600–1700 nm, QE ~90%, readout noise <175 e⁻) and uniform 808/980 nm excitation arrays enables high-sensitivity imaging in both NIR-I and NIR-II windows. The system includes an anesthesia and temperature-control platform, multiple exchangeable filters, an optional hyperspectral detection module, and supports dynamic imaging up to 120 fps, ideal for pharmacokinetics and molecular distribution studies. In our experiments, PEG-UCNPs and cMApt-UCNPs were administered via intravenous injection and imaged at 24, 48, and 72 hours using 980 nm excitation, with upconversion signals collected around ~700 nm for diagnostic evaluation.

*2.9 Cell viability and toxicity tests:* Alamar Blue is a non-toxic, water-soluble redox indicator used to assess cell viability by detecting the reduction of its oxidized blue form to a red, fluorescent state (Ex/Em: 560/590 nm; absorbance: 570/600 nm). For biocompatibility assessment, FaDu cells were seeded in 96-well plates (5,000 cells/well) and cultured for 24 hours. PEG-UCNPs or cMApt-UCNPs were added at concentrations of 15.625–1000 μg/mL and incubated for 24 or 48 hours. After adding 10% Alamar Blue to the medium, cells were incubated for 4 hours, and absorbance was measured (540/590 nm) using an ELISA reader.For therapeutic evaluation, FaDu cells were seeded similarly and treated with radioactive PEG-UCNP+NR or cMApt-UCNP+NR (15.625–1000 μg/mL). After 12 hours, cells were washed with PBS, replaced with fresh medium, and incubated for an additional 12–36 hours before Alamar Blue measurement. Data were analyzed using GraphPad Prism 8.

*2.10 Animal studies: In vivo* experiments were approved by the Institutional Animal Care and Use Committee (IACUC) of National Yang Ming Chiao Tung University (protocol no. 1140102). Five-week-old male nude mice (BALB/c nude) were used for upconversion imaging, therapeutic efficacy evaluation, biodistribution studies with a gamma counter, SPECT imaging, and H&E staining. Orthotopic tumor models were established by injecting 2 × 10^5^ FaDu cells (Matrix Gel:PBS = 1:1) into the right buccal mucosa. Experiments were initiated once the tumor volume reached approximately 100 mm^3^. For these studies, 100 µL (40 mg/kg) of PEG-UCNP, cMApt-UCNP, PEG-UCNP+NR, and cMApt-UCNP+NR samples were administered via subcutaneous or intravenous injection (n = 3 per group for quantitative analysis).

*2.11 In Vivo imaging system; IVIS:* An *in vivo* imaging system (IVIS, PerkinElmer) was used for non-invasive monitoring of tumor growth and nanoparticle biodistribution in mice. FaDu-GL cells stably expressing luciferase were subcutaneously implanted into the right buccal region of nude mice. Before imaging, mice were injected intraperitoneally with D-luciferin (150 mg/kg) and anesthetized with 2% isoflurane. Bioluminescent signals were captured using an open filter with a 1–3 minute exposure and analyzed using Living Image® software to quantify signal intensity (p/s/cm²/sr) and monitor tumor progression over time.

*2.12 Tissue staining assay:* After the *in vivo* experiments, mice were sacrificed at the designated time points, and tumors along with major organs (heart, liver, spleen, lung, kidney, and brain) were harvested and fixed in 4% paraformaldehyde for 24 hours. The tissues were subsequently dehydrated through a graded ethanol series, embedded in paraffin, and sectioned at 4–5 μm thickness. For hematoxylin and eosin (H&E) staining, the tissue sections were deparaffinized in xylene, rehydrated through a descending ethanol series, and stained with hematoxylin for 5 minutes, followed by eosin for 2 minutes. After staining, the sections were dehydrated, mounted with neutral resin, and imaged using an optical microscope. H&E staining was performed to evaluate tissue morphology and identify any pathological changes in tumors and major organs, thereby assessing potential systemic toxicity. immunohistochemistry (IHC), tumor sections underwent antigen retrieval by heating in citrate buffer (pH 6.0) for 10 minutes, followed by blocking with 5% bovine serum albumin (BSA) for 30 minutes. The sections were then incubated overnight at 4 °C with primary antibodies targeting Ki67 (a marker of cell proliferation) and Caspase-3 (a marker of apoptosis). After washing, the sections were treated with HRP-conjugated secondary antibodies for 1 hour at room temperature. Color development was achieved using a DAB substrate, and nuclei were counterstained with hematoxylin. Finally, the sections were dehydrated, mounted, and observed under a light microscope. Ki-67 and Caspase-3 staining enabled assessment of cell proliferation and apoptosis in tumor tissues. Additionally, H&E staining of major organs (heart, liver, spleen, lung, kidney, and brain) was conducted to examine overall tissue health and evaluate potential off-target toxicity following cMApt-UCNP treatment.

**3. Supplementary figures**

**Figure S1.** SAED pattern of NaLuF₄:Yb,Tm upconversion nanoparticles.

**Figure S2.** Quantitative analysis of fluorescence intensity. (a) Upconversion luminescence images of PBS, PEG-UCNPs, and cMApt-UCNPs under 980 nm excitation.(b–d) Corresponding pixel intensity profiles of PBS, PEG-UCNPs, and cMApt-UCNPs, respectively.

**Figure S3.** Characterization of nanoparticles by upconversion spectrum. (a) Upconversion fluorescence of cMApt-UCNPs dispersed in PBS under 980 nm laser excitation (20 W, 40%), recorded at 0, 10, 20, 30, 60, and 120 min, showing stable photoluminescence over time. (b) Upconversion fluorescence of PEG-UCNPs and cMApt-UCNPs embedded in 1% agarose under 980 nm laser excitation (20 W, 40%), recorded at the same time intervals, further confirming photostability within a gel matrix. (c) Upconversion fluorescence of PEG-UCNPs , cMApt-UCNPs, and cMApt-UCNP +NR embedded in 1% agarose under 980 nm laser excitation (20 W, 40%), recorded at the same time intervals, further confirming photostability within a gel matrix.

**Figure S4.** NanoDrop analysis of aptamer-conjugated UCNPs before and after neutron irradiation. (a) Spectra of D.D water, PEG-UCNPs, and cMApt-UCNPs, (b) Spectra of D.D water and neutron-irradiated cMApt-UCNPs (cMApt-UCNP + NR), (c) Quantitative NanoDrop analysis.

**Figure S5.** *In vitro* evaluation of cMApt-UCNPs. (a) Western blot analysis showing c-Met expression in oral cancer cells after incubation with PEG-UCNPs and cMApt-UCNPs for 24–72 hr. (b) Quantitative analysis of c-Met expression following treatment with PEG-UCNPs and cMApt-UCNPs. (c) c-Met expression levels in individual oral cancer cells. (d) Quantitative analysis of cleaved Caspase-3 expression, indicating apoptosis induced by cMApt-UCNP treatment. All experiments were performed with n = 3. Statistical significance was analyzed using multiple t-test (*p < 0.05, **p < 0.01, ***p < 0.001).

**Figure S6.** Cell uptake of PEG-UCNP and cMApt-UCNP (100 μg/mL) for 12 hr. Scale bar: 70 μm

**(a)**

**(b)**

**Figure S7.** Three-dimensional confocal laser scanning microscopy (CLSM) images showing cellular uptake of UCNP formulations. FaDu cells were incubated with (a) PEG-UCNPs and (b) cMApt-UCNPs for the indicated times, followed by staining with DAPI (blue, nuclei), UCNPs (green, nanoparticles), and c-Met (red, aptamers). The green fluorescence corresponds to the internalized UCNPs. Orthogonal and 3D reconstructed views demonstrate that cMApt-UCNPs exhibited markedly higher cellular uptake compared to UCNPs and PEG-UCNPs, with strong intracellular localization within the cytoplasmic region.

**Figure S8.** Confocal fluorescence images of the competitive binding assay in FaDu cells. Cells were pre-incubated with free c-Met aptamer (100-300 nM) before the addition of 150 μg/ml Cy5.5-labeled cMApt-UCNPs. Cell nuclei were stained with DAPI (blue), the Cy5.5-labeled aptamer is shown in red, and UCNP fluorescence is shown in green. Scale bar: 10 μm

**Figure S9.** Competitive inhibition assay of cMApt-UCNP uptake in FaDu cells. FaDu cells were pretreated (a) without, with (b) 100 or (c) 300 nmol free c-Met aptamer for 4 h, followed by incubation with Cy5.5-labeled cMApt-UCNPs (150 μg/mL) for 9 h.

**Figure S10.** Cell viability of OMF cells treated with PEG-UCNPs (a, c) or cMApt-UCNPs (b, d) for 24 and 48 h. Panels (a) and (b) present the data as a function of nanoparticle mass concentration, whereas panels (c) and (d) show the same datasets plotted against Lu^3+^ molar concentration. All experiments were performed with n = 5. Statistical significance was analyzed using one-way ANOVA with Tukey’s post hoc test (*p < 0.05, **p < 0.01, ***p < 0.001).

**Figure S11.** The same experimental data as shown in Figure 4d, replotted using different x-axis representations. Cell viability of FaDu cells treated with PEG-UCNPs or cMApt-UCNPs for 24 and 48 h. Present the same datasets plotted against Lu^3+^ molar concentration. All experiments were performed with n = 5. Statistical significance was analyzed using one-way ANOVA with Tukey’s post hoc test (*p < 0.05, **p < 0.01, ***p < 0.001).

**Figure S12.** Colony formation of FaDu cells after 12 hr incubation with PBS/PEG-UCNP/cMApt-UCNP/PEG-UCNP+NR/cMApt-UCNP+NR (Non-irradiation 100 μg/mL, Irradiation 100 μg/mL, 100μCi) and incubated for 5 Days. Quantitative data of cell populations in different groups were obtained using ImageJ. All experiments were performed with n = 5. Statistical significance was analyzed using Multiple t test (*p < 0.05, **p < 0.01, ***p < 0.001).

**Figure S13.** Animal experiment flow chart.

**Figure S14.** *In vivo* upconversion fluorescence imaging of tumor-bearing mice injected with PEG-UCNP or cMApt-UCNP at 24, 48, and 72 hours post-injection, with or without 980 nm laser excitation.

**Figure S15.** Comparison of SPECT/CT images in axial and sagittal planes. The axial view highlights the horizontal distribution of the tracer within the tumor and surrounding tissues, whereas the sagittal view provides a longitudinal perspective, revealing 177Lu-UCNP tracer (a) PEG-UCNP and (b) cMApt-UCNPs uptake along the anterior-posterior axis.

**Figure S16.** Representative SPECT/CT images acquired at (a) 4, (b) 24, and (c) 48 h after intravenous administration of ^177^Lu-labeled cMApt-UCNPs in FaDu tumor-bearing mice.

**Figure S17.** Individual tumor growth curves of tumor-bearing mice in each treatment group. Each line represents tumor volume progression in a single mouse over the treatment period. While tumors in the control and PEG-UCNP+NR groups exhibited heterogeneous but continuous growth, mice treated with cMApt-UCNP+NR showed consistently suppressed tumor progression, indicating a robust and reproducible therapeutic effect. All experiments were performed with n = 5.

**Figure S18.** Hematoxylin and eosin (H&E) staining of major organs from the control, PEG-UCNP + NR, and cMApt-UCNP + NR groups. Representative sections of the heart, liver, spleen, lung, and kidney showed generally preserved histological architecture without obvious signs of tissue damage, inflammation, or necrosis following treatment, suggesting no evident treatment-associated histological toxicity under the experimental conditions. Scale bars: 50 μm

**4. Supplementary tables**

**Table S1.** DLS analysis of UCNPs, PEG-UCNPs, and cMApt-UCNPs. The table summarizes the average hydrodynamic diameters and zeta potential values for each formulation. Data are presented as mean ± PDI (n = 3).

**Table S2.** Quantitative biodistribution analysis of neutron-activated PEG-UCNPs and cMApt-UCNPs in tumor, spleen, and liver tissues. Radioactivity was measured as kBq/cc. The PEG-UCNP + NR group exhibited T/S and T/L ratios of 1.012 and 0.98, respectively, whereas the cMApt-UCNP + NR group showed markedly increased T/S and T/L ratios of 13.87 and 10.95, respectively. Data are presented as mean ± SD. All experiments were performed with n = 3.

**Table S3.** Biodistribution analysis of radioactivity in blood at 72, 96, and 120 h after intravenous administration of cMApt-UCNPs-^177^Lu. Radioactivity was quantified by gamma counting and expressed as CPM, %ID, and %ID/g based on the measured blood weight.
